# Supplementary material for: CSF1R inhibitors induce a sex-specific resilient microglial phenotype and functional rescue in a tauopathy mouse model
Source: Nat Commun. 2023 Jan 9;14:118. doi: 10.1038/s41467-022-35753-w (PMC9829908; doi:10.1038/s41467-022-35753-w)
Supplement: Supplementary file 1 — Supplementary Information [file 41467_2022_35753_MOESM1_ESM.pdf]

SUPPLEMENTARY INFORMATION

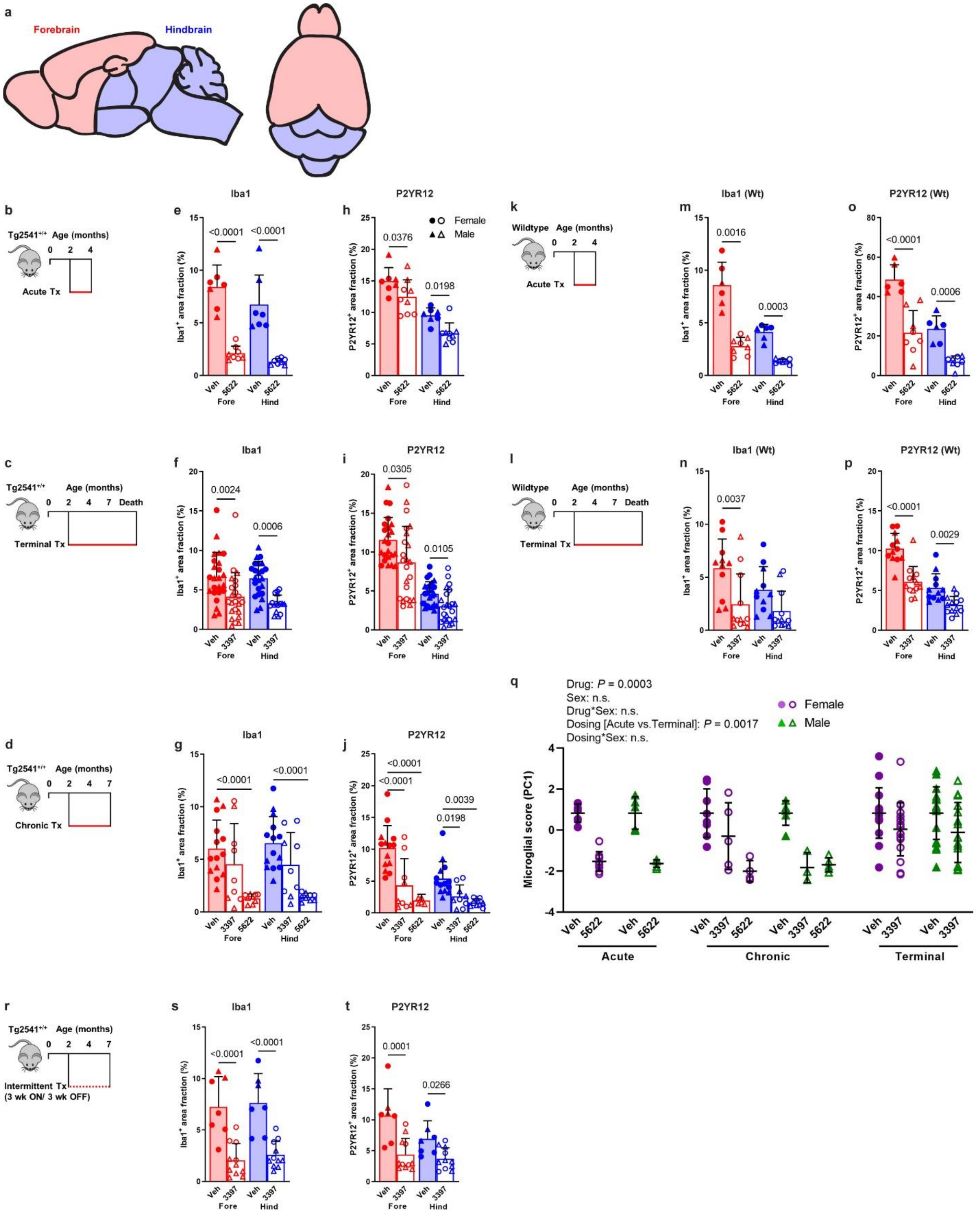

Supplementary Fig. 1| Microglial depletion by CSF1R inhibitors in Tg2541 and wild type

mice. a, Sagittal and superior diagrams of the mouse brain divided into two regions: the forebrain, containing the cortex, hippocampus, striatum, and olfactory bulb; and the hindbrain, containing the thalamus, hypothalamus, midbrain, cerebellum, and brain stem. **b−d**, Schematics of acute (**b**), terminal (**c**), or chronic (**d**) PLX treatment of Tg2541 mice from 2−4 mo of age, 2 mo of age until death, or 2−7 mo of age, respectively. **e−g**, Quantification of the Iba1-positive area fraction by IHC in the forebrains or hindbrains of Tg2541 mice receiving acute (**e**), terminal (**f**), or chronic (**g**) treatment with vehicle, PLX3397 (275 mg/kg oral), or PLX5622 (1200 mg/kg oral). **h−j**, Quantification of the P2yr12-positive area fraction by IHC in the forebrains or hindbrains of Tg2541 mice receiving acute (**h**), terminal (**i**), or chronic (**j**) treatment with vehicle, PLX3397, or PLX5622. **k**, **l**, Schematics of acute (**k**) or terminal (**l**) PLX treatment of C57BL/6J wild type mice (Wt) from 2−4 mo of age, or 2 mo of age until death, respectively. **m**, **n**, Quantification of the Iba1-positive area fraction by IHC in the forebrain and hindbrain of Wt mice receiving acute (**m**) or terminal (**n**) treatment with vehicle, PLX3397, or PLX5622. **o**, **p**, Quantification of the P2yr12-positive area fraction by IHC in the forebrains or hindbrains of Wt mice receiving acute (**o**) or terminal (**p**) treatment with vehicle, PLX3397, or PLX5622. *P* values for all statistically significant differences ($P < 0.05$) are shown. **q**, Principal component analysis was performed, using all data presented in **e−j** and **m−p** to calculate a ‘microglial score’ that represents the amount of Iba1 and P2yr12 staining in both the forebrains and hindbrains of Tg2541 and Wt mice. All data was first standardized to the respective vehicle-treated group of the same sex and same dosing paradigm. Then, two principal components (PC1 and PC2) were identified which accounted for 79.0% of the total variance in the data. Multiple linear regression was performed on PC1 of the drug-treated groups to evaluate the main effects sex and dosing paradigm, and the dosing*sex interaction effect. Multiple linear regression was performed on all groups to determine the main effect of drug and the drug*sex interaction effect. *P* values for all statistically significant differences ($P < 0.05$) are shown. n.s. indicates not statistically significant. PC2 was also evaluated, but only the drug main effect was statistically significant. **r**, Schematic of intermittent PLX treatment of Tg2541 mice from 2−7 mo of age, with three weeks on treatment followed by three weeks off of treatment. **s**, **t**, Quantification of the Iba1-positive (**s**) or P2yr12-positive (**t**) area fractions by IHC in the forebrains or hindbrains of Tg2541 mice receiving intermittent treatment with vehicle or PLX3397. In **e−j**, **m−p**, **s**, and **t**, each symbol represents the forebrain or hindbrain of an individual mouse, with female mice shown as closed or open circles and male mice shown as closed or open triangles. Error bars represent s.d. of the mean. Two-way ANOVA with Holm-Šidák post hoc testing was used in **e**, **f**, **h**, **i**, **m−p**, **s**, and **t**. Welch ANOVA with Dunnett T3 post hoc testing was used in **g** and **j**.

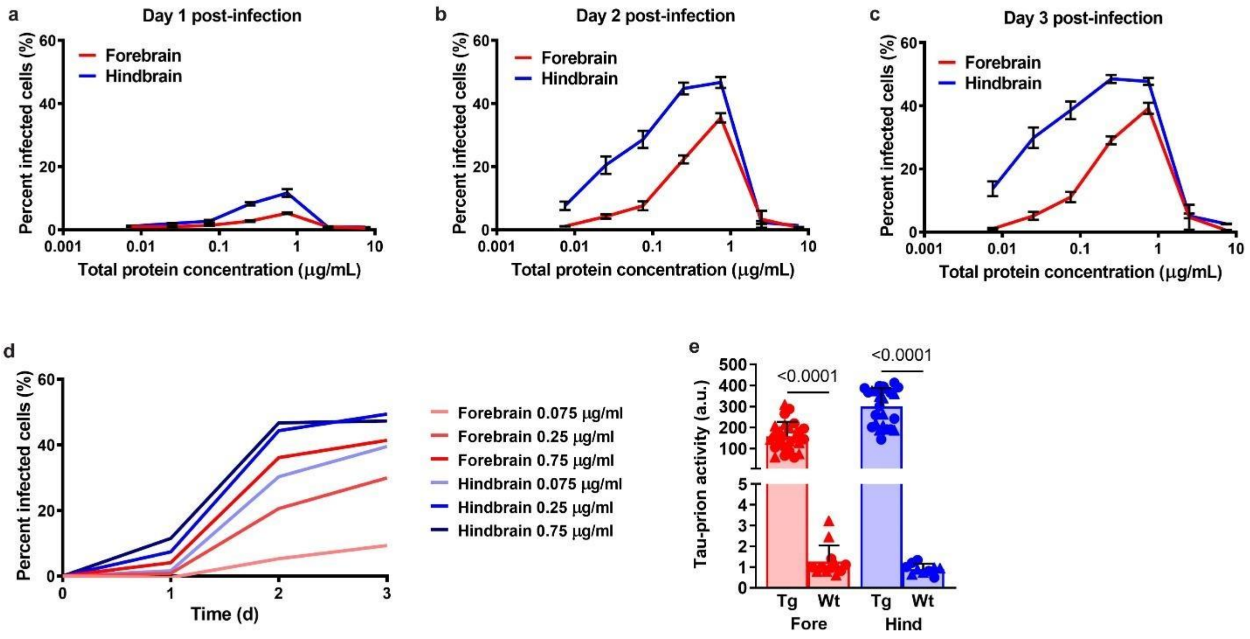

Supplementary Fig. 2| Optimization of HEK293T cell bioassay for measuring tau-prions in Tg2541 mouse brain homogenates. a−c, Percent of HEK293T cells expressing YFP-tau-RD*P301L/V337M with tau aggregates at one (**a**), two (**b**), or three (**c**) days post-infection with Tg2541 mouse forebrain or hindbrain homogenates at concentrations ranging from 0.0075−7.5 µg/ml. Error bars represent s.e.m. of three terminal Tg2541 mice. **d**, Percent of cells infected with tau aggregates over time following infection with 0.075, 0.25, or 0.75 µg/ml Tg2541 mouse forebrain or hindbrain homogenates. Lines represent the means of three terminal Tg2541 mice. **e**, Tau-prion levels in 0.25 µg/ml forebrain or hindbrain homogenates of Tg2541 (Tg) or wild type (Wt) mice, measured using the optimized HEK293T cell tau-prion bioassay. Each symbol represents the forebrain or hindbrain of an individual mouse and female mice are shown as closed circles while male mice are shown as closed triangles. Two-way ANOVA with Holm-Šidák post hoc testing was used and *P* values for all statistically significant differences (*P* < 0.05) are shown.

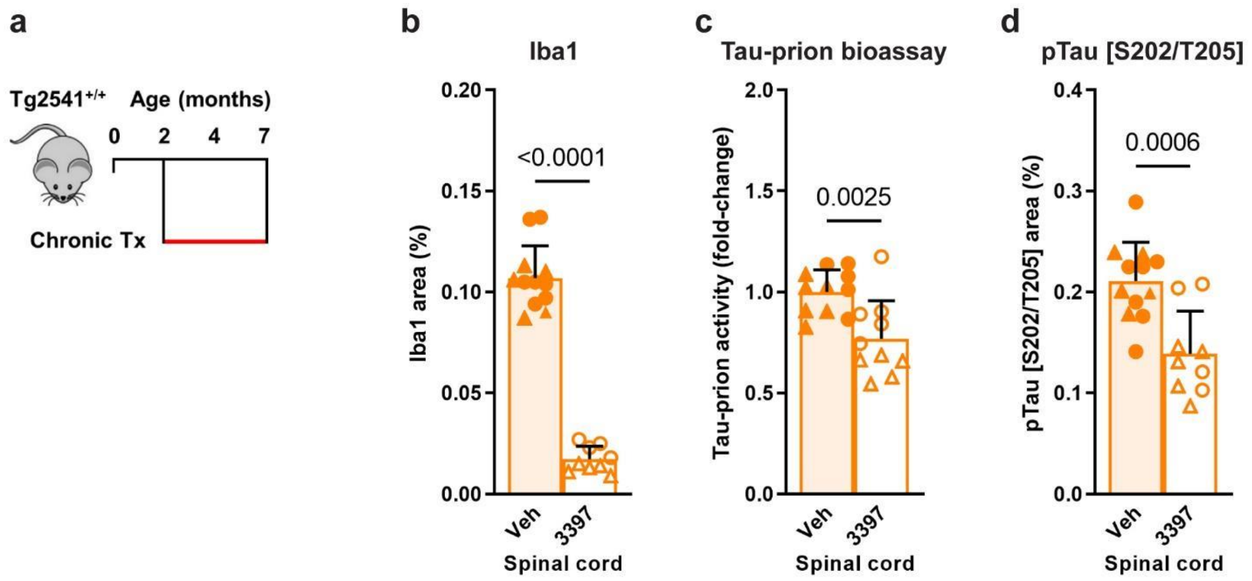

Supplementary Fig. 3| CSF1R inhibition reduces microglia and pathogenic tau levels in the spinal cords of Tg2541 mice. a, Schematic of chronic PLX treatment of Tg2541 mice from 2−7 mo of age. **b**, Quantification of the Iba1-positive area fraction by IHC in the spinal cords of Tg2541 mice receiving chronic treatment with vehicle or PLX3397 (275 mg/kg oral). **c**, Tau-prion levels in spinal cord tissue homogenates of Tg2541 mice receiving chronic treatment with vehicle or PLX3397, measured using the HEK293T cell tau-prion bioassay and normalized to the vehicle group. **d**, Quantification of pTau [S202/T205]-positive area by IHC in the spinal cords of Tg2541 mice receiving chronic treatment with vehicle or PLX3397. In **b−d**, each symbol represents an individual mouse, with female mice shown as closed or open circles and male mice shown as closed or open triangles. Error bars represent s.d. of the mean. Unpaired t tests were used and *P* values for all statistically significant differences ($P < 0.05$) are shown.

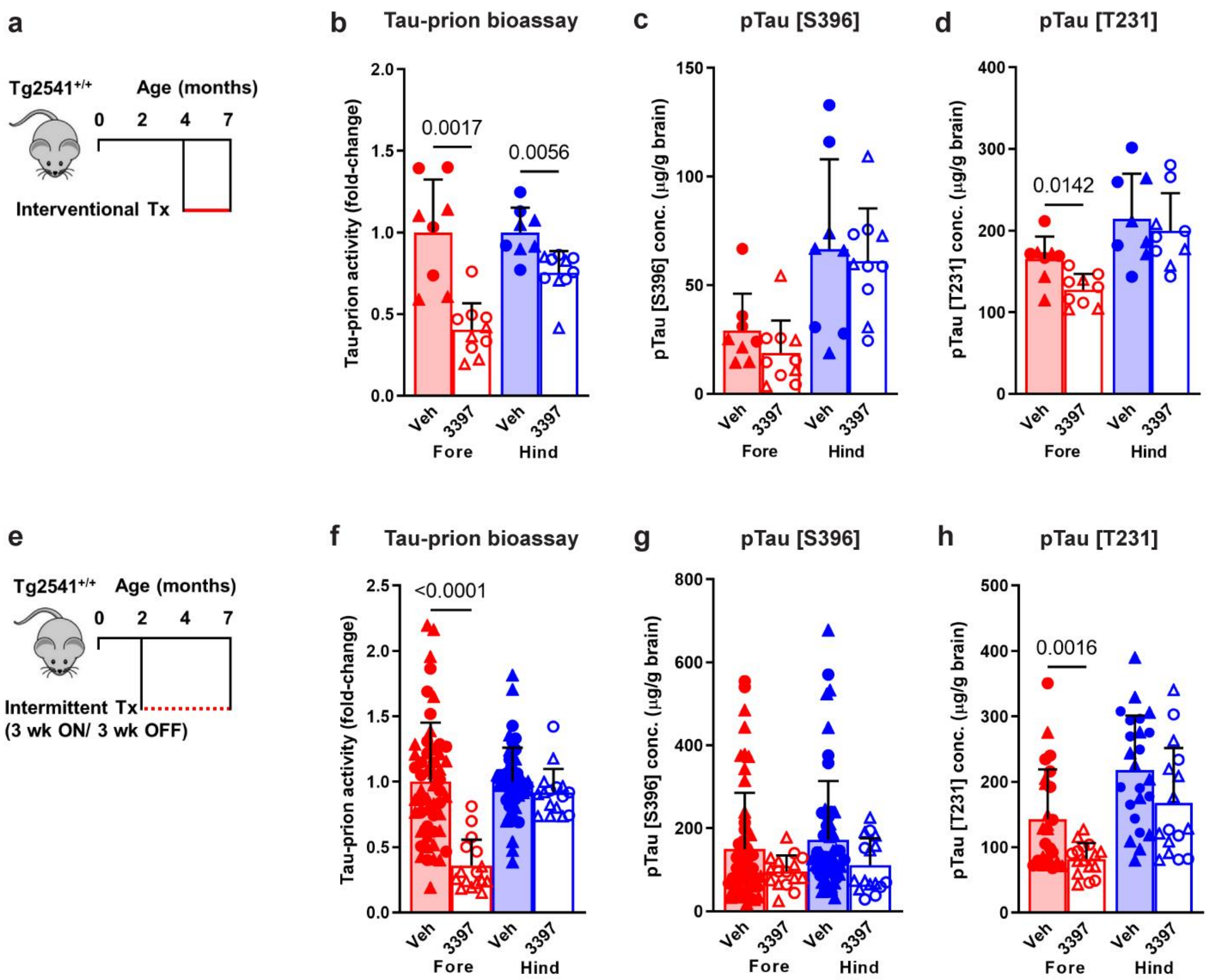

Supplementary Fig. 4| Interventional or intermittent CSF1R inhibition reduces pathogenic tau levels in the brains of Tg2541 mice. a, Schematic of interventional PLX treatment of Tg2541 mice from 4−7 mo of age. **b**, Tau-prion levels in forebrain and hindbrain tissue homogenates of Tg2541 mice receiving interventional treatment with vehicle or PLX3397 (275 mg/kg oral), measured using the HEK293T cell tau-prion bioassay and normalized to the vehicle group. **c**, Levels of pTau [S396] measured by ELISA in formic acid extracts of forebrain and hindbrain tissue homogenates of Tg2541 mice receiving interventional treatment with vehicle or PLX3397, normalized to total protein concentration. **d**, Levels of pTau [T231] measured by ELISA in formic acid extracts of forebrain and hindbrain tissue homogenates of Tg2541 mice receiving interventional treatment with vehicle or PLX3397, normalized to total protein concentration. **e**, Schematic of intermittent PLX treatment of Tg2541 mice from 2−7 mo of age, with three weeks on treatment followed by three weeks off of treatment. **f**, Tau-prion levels in forebrain and hindbrain tissue homogenates of Tg2541 mice receiving intermittent treatment

with vehicle or PLX3397, measured using the HEK293T cell tau-prion bioassay and normalized to the vehicle group. **g**, Levels of pTau [S396] measured by ELISA in formic acid extracts of forebrain and hindbrain tissue homogenates of Tg2541 mice receiving intermittent treatment with vehicle or PLX3397, normalized to total protein concentration. **h**, Levels of pTau [T231] measured by ELISA in formic acid extracts of forebrain and hindbrain tissue homogenates of Tg2541 mice receiving intermittent treatment with vehicle or PLX3397, normalized to total protein concentration. In **b−d** and **f−h**, each symbol represents the forebrain or hindbrain of an individual mouse, with female mice shown as closed or open circles and male mice shown as closed or open triangles. Error bars represent s.d. of the mean. Two-way ANOVA with Holm-Šidák post hoc testing was used and *P* values for all statistically significant differences ($P < 0.05$) are shown.

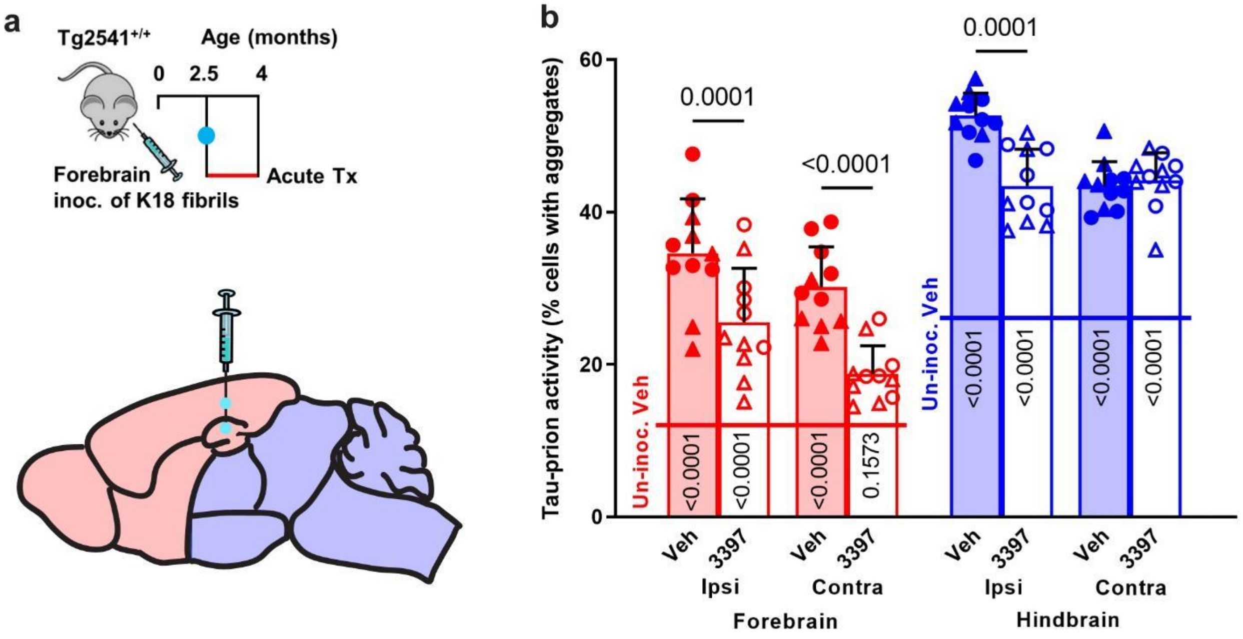

Supplementary Fig. 5| CSF1R inhibition reduces pathogenic tau spreading following K18 forebrain inoculation. a, Schematic of acute PLX treatment of Tg2541 mice from 2.5−4 mo of age following inoculation of K18 tau fibrils into the hippocampus and overlying cortex (forebrain regions) at 2.5 mo of age. **b**, Tau-prion levels in the ipsilateral (inoculated side) or contralateral (un-inoculated side) forebrain or hindbrain tissue homogenates of Tg2541 mice receiving acute treatment with vehicle or PLX3397 (275 mg/kg oral) following K18 forebrain inoculation, measured using the HEK293T cell tau-prion bioassay and presented as percent of cells with tau aggregates. Horizontal lines across bars indicate the mean tau-prion level in forebrain (12.54% cells with aggregates) or hindbrain (26.06% cells with aggregates) tissue homogenates of Tg2541 mice that did not undergo K18 inoculation and received acute treatment with vehicle (Un-inoc. Veh). Each symbol represents the ipsilateral or contralateral forebrain or hindbrain of an individual mouse, with female mice shown as closed or open circles and male mice shown as closed or open triangles. Error bars represent s.d. of the mean. Three-way ANOVA with Holm-Šidák post hoc testing was used and *P* values for all statistically significant differences ($P < 0.05$) between Veh and 3397 are shown above the bars. *P* values for all differences between each group and the respective brain region of Un-inoc. Veh mice are shown on the bars.

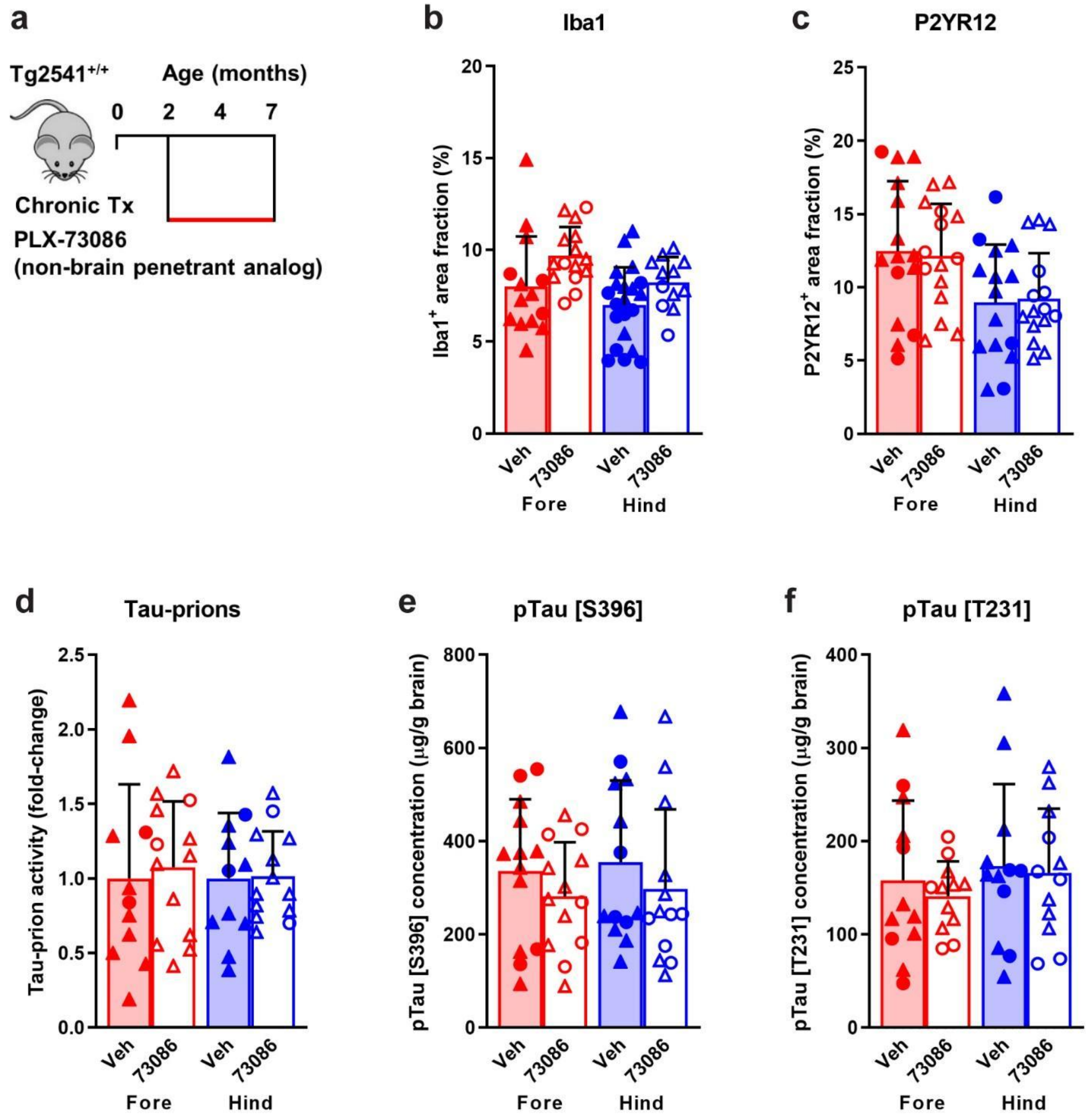

Supplementary Fig. 6| Non-brain penetrant analog of CSF1R inhibitors does not reduce microglia or pathogenic tau levels in the brains of Tg2541 mice. a, Schematic of chronic treatment of Tg2541 mice from 2−7 mo of age with PLX73086, a non-brain penetrant analog of PLX3397 and PLX5622. **b**, **c**, Quantification of the Iba1-positive (**b**) or P2yr12-positive (**c**) area fractions by IHC in the forebrains or hindbrains of Tg2541 mice receiving chronic treatment with vehicle or PLX73086 (200 mg/kg oral). **d**, Tau-prion levels in forebrain and hindbrain tissue homogenates of Tg2541 mice receiving chronic treatment with vehicle or PLX73086, measured

using the HEK293T cell tau-prion bioassay and normalized to the vehicle group. **e**, **f**, Levels of pTau [S396] (**e**) or pTau [T231] (**f**) measured by ELISA in formic acid extracts of forebrain and hindbrain tissue homogenates of Tg2541 mice receiving chronic treatment with vehicle or PLX73086, normalized to total protein concentration. In **b−f**, each symbol represents the forebrain or hindbrain of an individual mouse, with female mice shown as closed or open circles and male mice shown as closed or open triangles. Error bars represent s.d. of the mean. Two-way ANOVA with Holm-Šidák post hoc testing was used and *P* values for all statistically significant differences ($P < 0.05$) are shown.

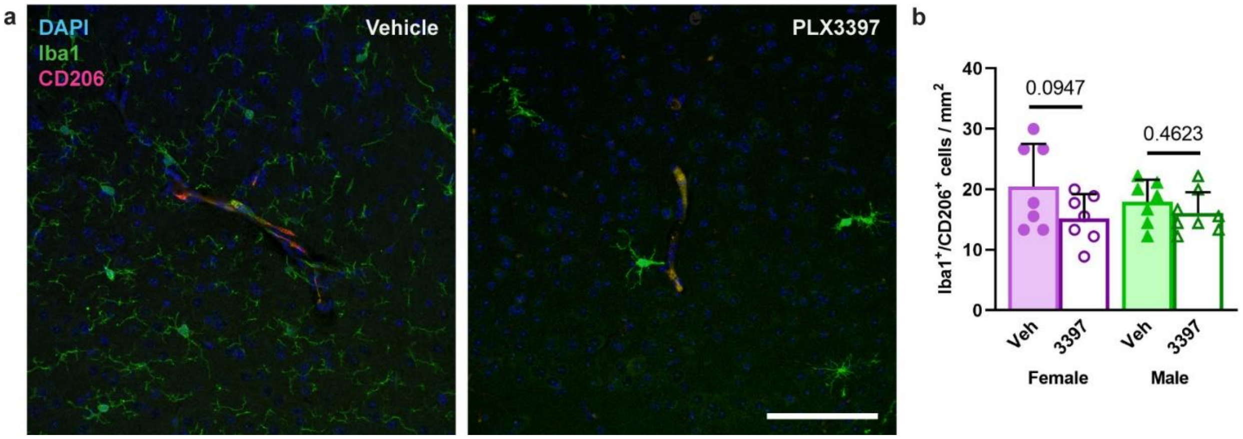

Supplementary Fig. 7| Pharmacological CSF1R inhibition does not deplete perivascular macrophages. a, Representative immunofluorescence images of Tg2541 mouse cortical blood vessels labeled for perivascular macrophages (Iba1$^+$/CD206$^+$) and microglia (Iba1$^+$/CD206$^-$). Iba1 labels both PVMs and microglia. Mice were treated with PLX3397 from 2.5 mo of age until death, following inoculation of K18 tau fibrils into the midbrain (hindbrain region) at 2.5 mo of age. Scale bar, 100 μm. **b**, Quantification of Iba1$^+$/CD206$^+$ cell density in images of Tg2541 mouse cortical blood vessels. Each symbol represents an individual mouse and is the average of six quantified blood vessel images from that mouse. Error bars represent s.d. of the mean. Two-way ANOVA was used and the *P* values for Holm-Šidák post hoc testing between treatment groups are shown.

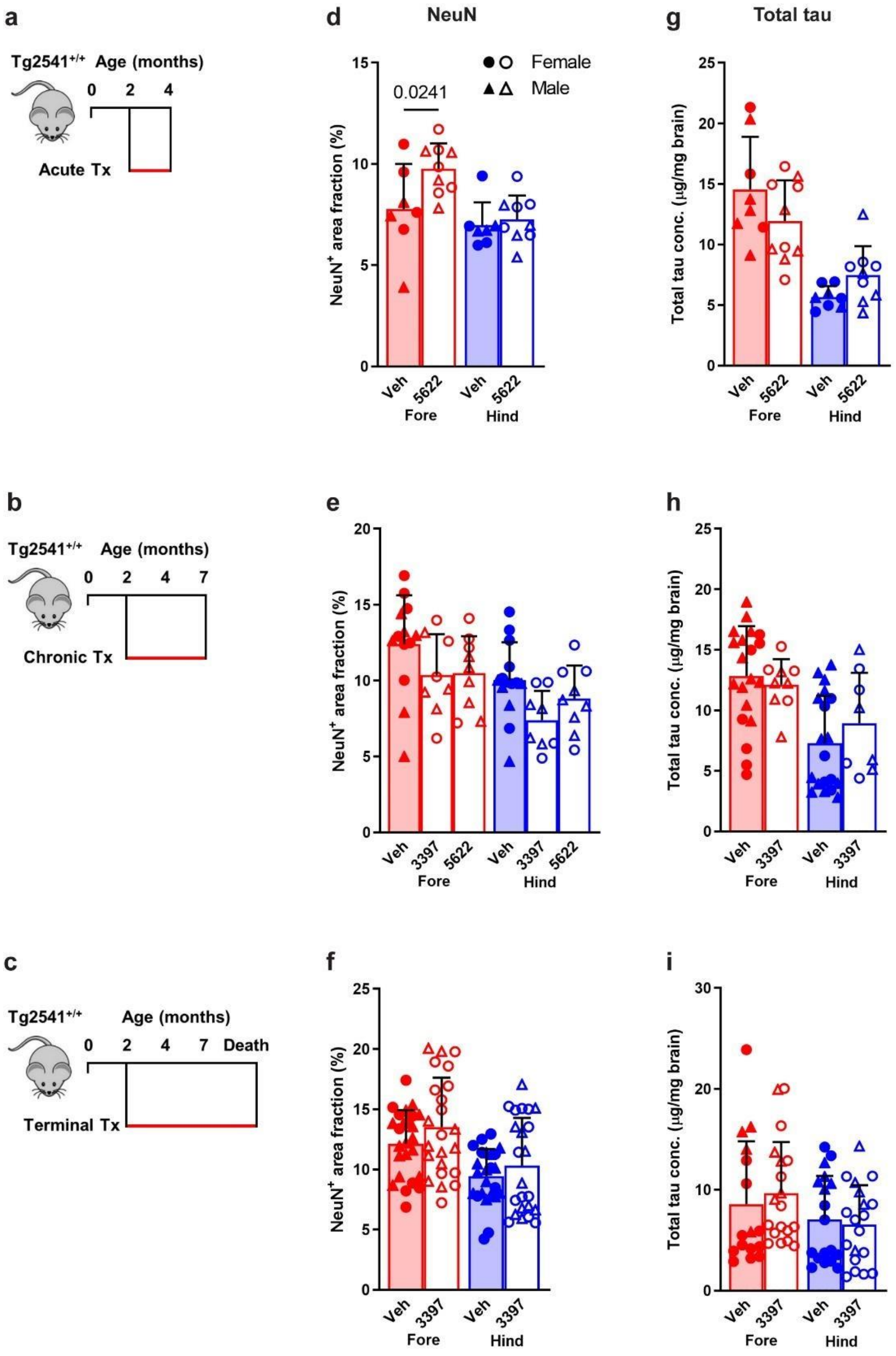

Supplementary Fig. 8| CSF1R inhibition by three treatment paradigms does not affect neurons or total tau levels. a−c, Schematics of acute (**a**), chronic (**b**), or terminal (**c**) PLX treatment of Tg2541 mice from 2−4 mo of age, 2−7 mo of age, or 2 mo of age until death, respectively. **d−f**, Quantification of neuronal nuclei (NeuN)-positive area fraction by IHC analysis

of forebrain and hindbrain areas of Tg2541 mice receiving acute (**d**), chronic (**e**), or terminal (**f**) treatment with vehicle, PLX3397 (275 mg/kg oral), or PLX5622 (1200 mg/kg oral). **g−i**, Levels of total tau measured by ELISA in forebrain and hindbrain tissue homogenates of Tg2541 mice receiving acute (**g**), chronic (**h**), or terminal treatment (**i**) with vehicle, PLX3397, or PLX5622, normalized to total protein concentration. In **d−i**, each symbol represents the forebrain or hindbrain of an individual mouse, with female mice shown as closed or open circles and male mice shown as closed or open triangles. Error bars represent s.d. of the mean. Two-way ANOVA with Holm-Šidák post hoc testing was used in **d**, and **f−i**. One-way ANOVA with Holm-Šidák post hoc testing was used in **e**. P values for all statistically significant differences ($P < 0.05$) are shown.

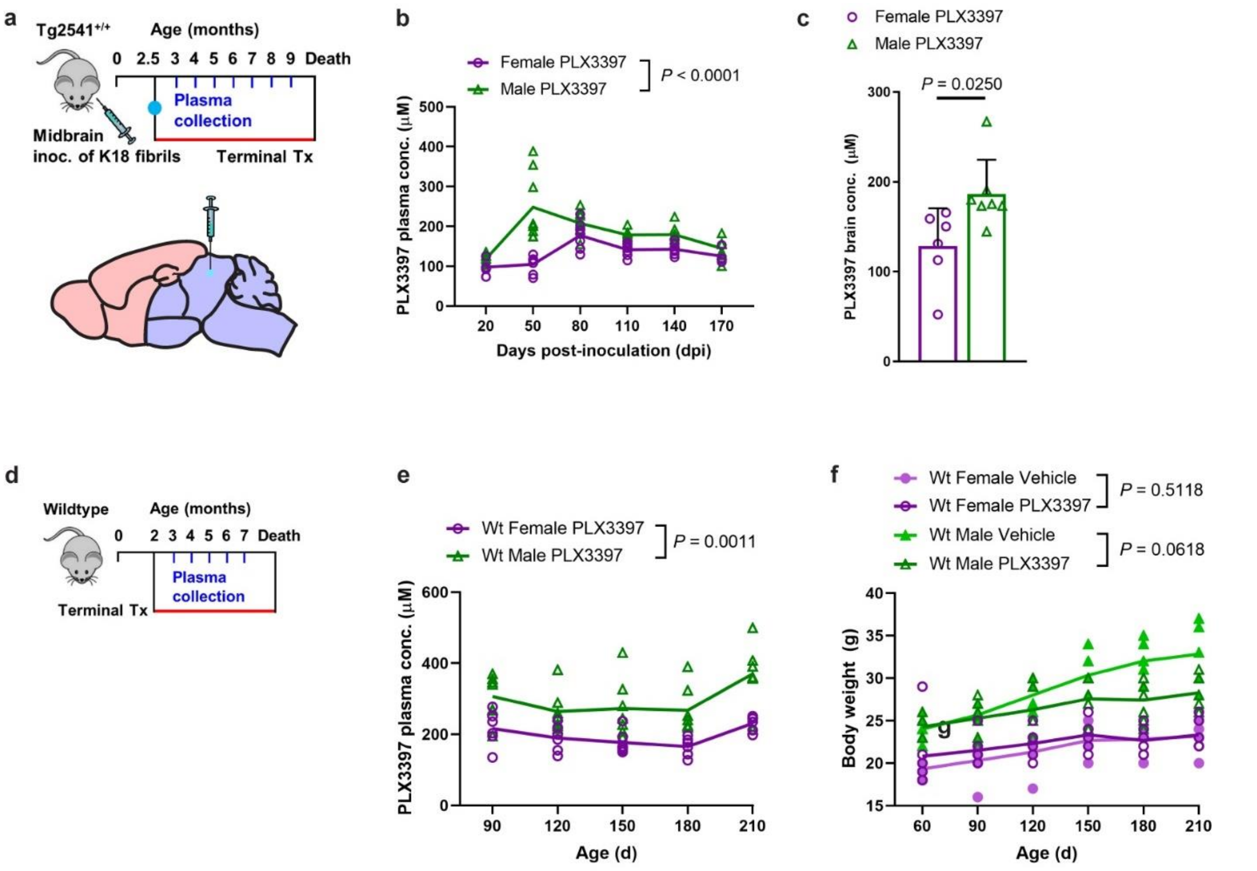

Supplementary Fig. 9| PLX3397 levels are increased in male Tg2541 and wild type mice. **a**, Schematic of terminal PLX3397 treatment of Tg2541 mice from 2.5 mo of age until death, following inoculation of K18 tau fibrils into the midbrain (hindbrain region) at 2.5 mo of age. Blood plasma was collected at monthly intervals from 3−9 mo of age. **b**, Plasma concentration of PLX3397 in female or male Tg2541 mice inoculated with K18 followed by terminal treatment with PLX3397, plotted over days post inoculation (dpi). Mixed-effects analysis (Restricted maximum likelihood) was used to compare female and male mice and the *P* value is shown. **c**, Brain concentration of PLX3397 at death in female or male Tg2541 mice inoculated with K18 followed by terminal treatment with PLX3397. Unpaired t test was used to compare female and male mice and the *P* value is shown. Each symbol represents an individual mouse and the error bars represent the s.d. of the mean. **d**, Schematic of terminal PLX treatment of C57BL/6J wild type mice (Wt) from 2 mo of age until death and blood plasma collected at 3, 4, 5, 6, and 7 mo of age. **e**, Plasma concentration of PLX3397 in female or male Wt mice. The difference between female and male mice was assessed by two-way repeated measures ANOVA and the *P* value is shown. **f**, Body weights of female or male Wt mice treated with vehicle or PLX3397. Differences in weight between vehicle and PLX3397 treatment in male or female mice were evaluated by

two-way repeated measures ANOVA and *P* values are shown. In **b**, **e**, and **f**, each symbol represents an individual mouse and the lines represent the group means.

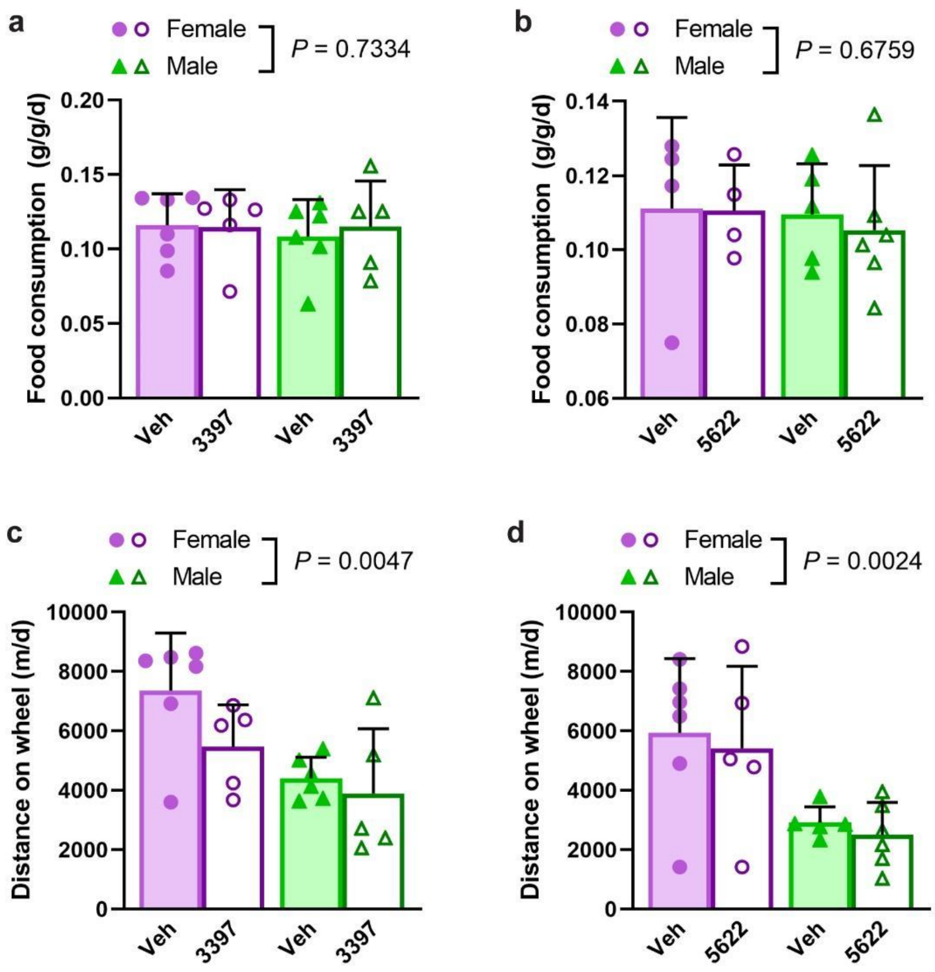

Supplementary Fig. 10| Food consumption and activity of Tg2541 mice treated with CSF1R inhibitors. a, **b**, Food consumption measured in Tg2541 mice receiving terminal treatment with vehicle or PLX3397 (275 mg/kg oral) (**a**), or vehicle or PLX5622 (1200 mg/kg oral) (**b**), reported as grams of food per gram of mouse body weight per day (g/g/d). **c**, **d**, Running wheel activity measured in Tg2541 mice receiving terminal treatment with vehicle or PLX3397 (**c**), or vehicle or PLX5622 (**d**), reported as total distance traveled in meters per day (m/d). In **a−d**, each symbol represents an individual mouse, with female mice shown as closed or open circles and male mice shown as closed or open triangles. Error bars represent s.d. of the mean. Two-way ANOVA was used to compare female and male mice and *P* values are shown.

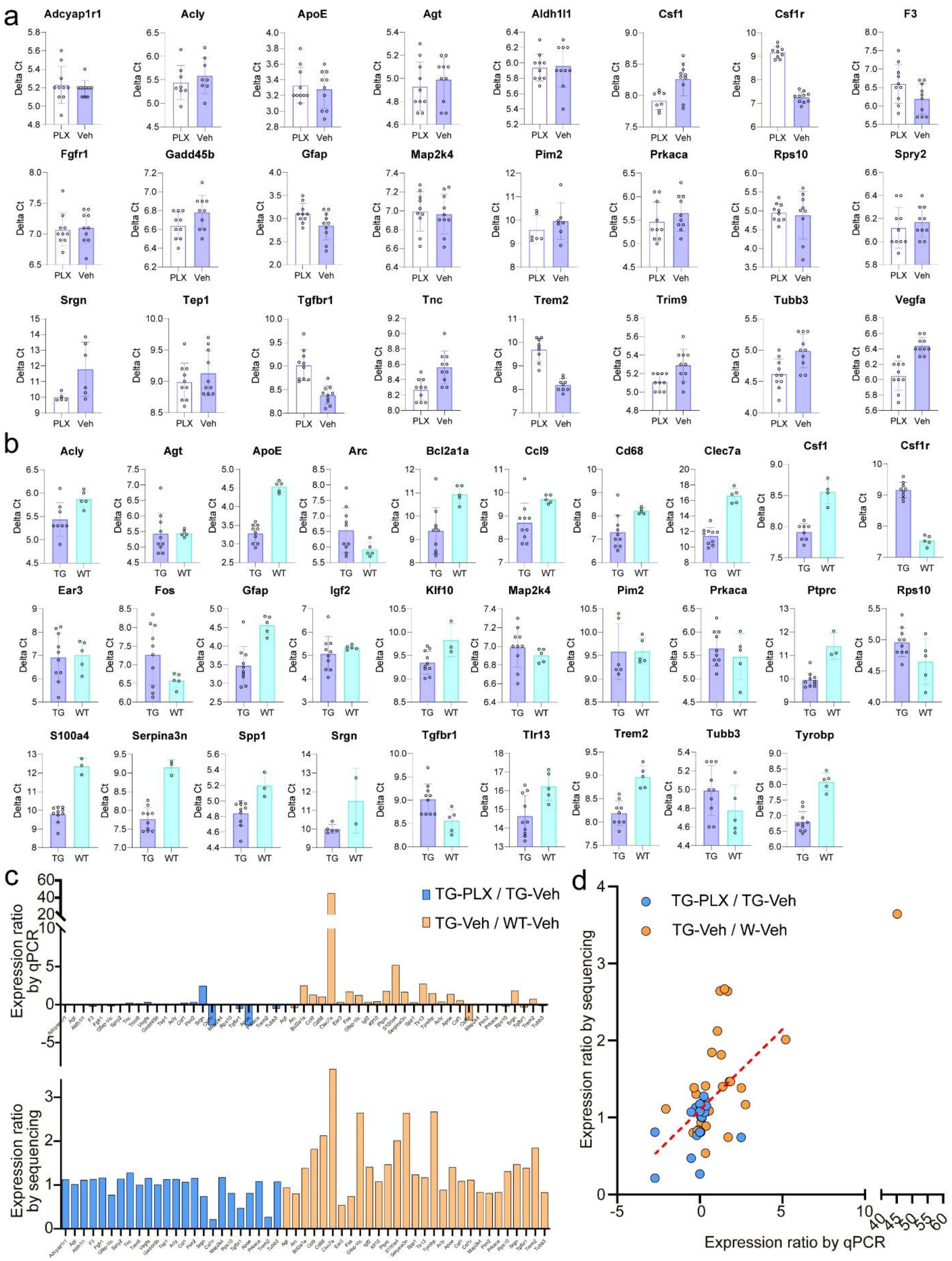

Supplementary Fig. 11| Validation of Nanostring sequencing results with real-time PCR.

a, Delta Ct values of 24 selected genes in hindbrains of Tg2541 mice treated with PLX5622

(1200 mg/kg) and vehicle. A lower Delta Ct reading indicates higher expression level. **b**, Delta Ct values of 29 selected genes in hindbrains of Tg2541 and wild type mice treated with vehicle. A lower Delta Ct reading indicates higher expression level. In **a** and **b**, data are presented as mean ± S.D. **c**, Expression ratios of the 53 genes in (**a**) and (**b**) as measured by qPCR and Nanostring sequencing. **d**, Scatter plot of the data in (**c**). Red dotted line shows a linear trend line. In **a**–**c,** n=10 mice in TG groups, and n=5 mice for WT groups. In **d**, n=24 genes for PLX vs. Veh group, and n=29 genes for TG vs. WT group.

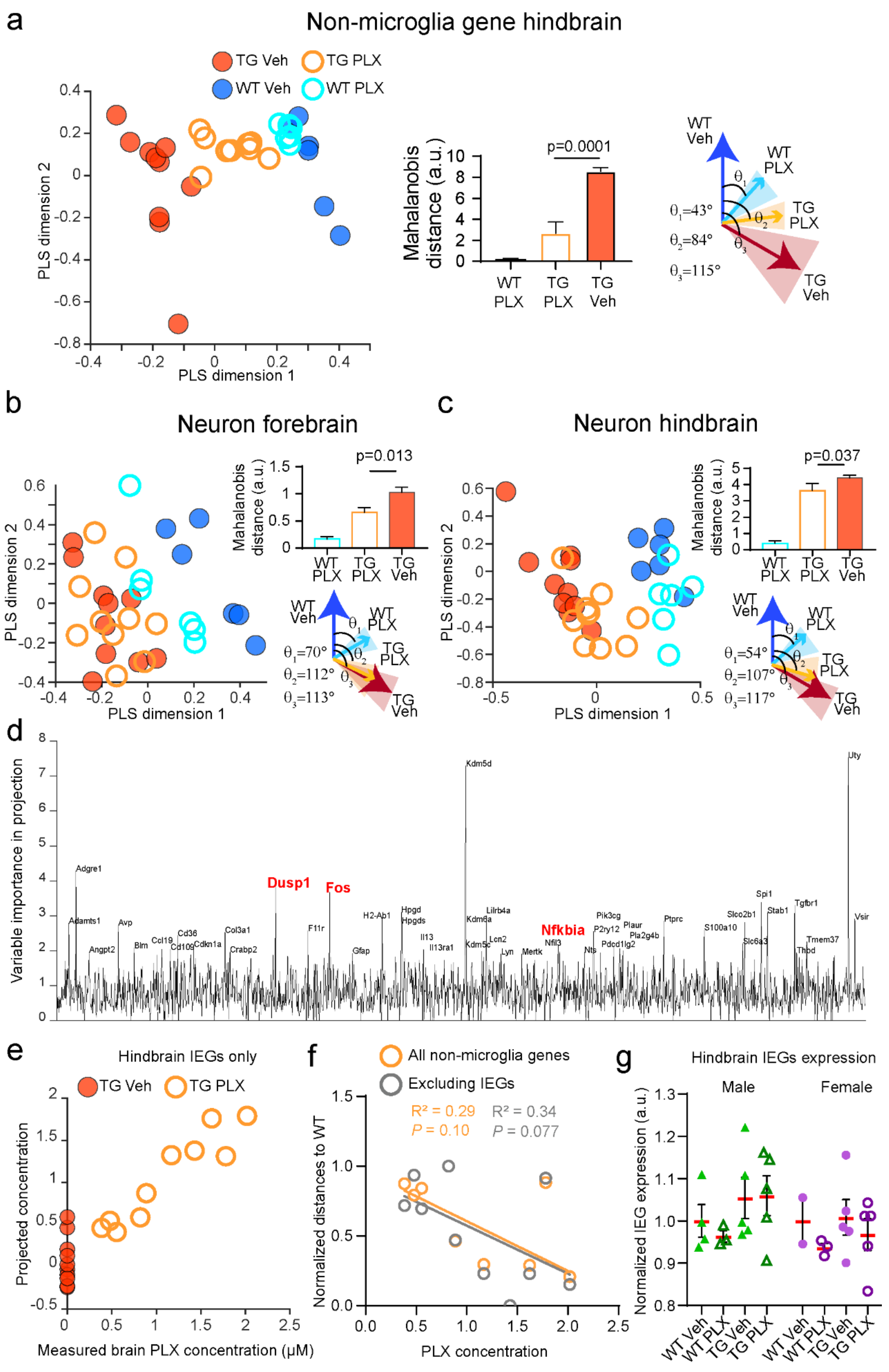
a
Non-microglia gene hindbrain
TG Veh
TG PLX
WT Veh
WT PLX
PLS dimension 2
PLS dimension 1
Mahalanobis distance (a.u.)
p=0.0001
WT PLX
TG PLX
TG Veh
WT Veh
θ1=43°
θ2=84°
θ3=115°
b
Neuron forebrain
p=0.013
θ1=70°
θ2=112°
θ3=113°
c
Neuron hindbrain
p=0.037
θ1=54°
θ2=107°
θ3=117°
d
Variable importance in projection
Adgre1
Adamts1
Angpt2
Avp
Blm
Ccl19
Cd36
Cd109
Cdkn1a
Col3a1
Crabp2
Dusp1
Fos
F11r
Gfap
H2-Ab1
Hpgd
Hpgds
Il13
Il13ra1
Kdm5d
Kdm6a
Kdm5c
Lilrb4a
Lcn2
Lyn
Mertk
Nfkbia
Nfil3
Nts
Pik3cg
P2ry12
Plaur
Pdcd1lg2
Pla2g4b
Ptprc
S100a10
Slco2b1
Slc6a3
Spi1
Stab1
Tgfbr1
Tmem37
Thbd
Uty
Vsir
e
Hindbrain IEGs only
TG Veh
TG PLX
Projected concentration
Measured brain PLX concentration (μM)
f
All non-microglia genes
Excluding IEGs
R² = 0.29
P = 0.10
R² = 0.34
P = 0.077
Normalized distances to WT
PLX concentration
g
Hindbrain IEGs expression
Male
Female
Normalized IEG expression (a.u.)
WT Veh
WT PLX
TG Veh
TG PLX
WT Veh
WT PLX
TG Veh
TG PLX

Supplementary Fig. 12| Gene expression pattern analyses of non-microglial and neuron-specific genes. **a−c,** Analyses similar to Fig. **6c−e**, using (**a**) all non-microglial genes in hindbrain, or neuron-specific genes in (**b**) forebrain and (**c**) hindbrain. **d−g**, Analyses similar to Fig. 6g−j, using data from hindbrains. In **a-c**, data are presented as mean ± S.D., with n=10 mice for TG groups, and n=6 mice for WT groups. Mann-Whitney tests were used for comparison. In **g**, n=4, 3, 5, 5, 2, 3, 5, 5 mice for each group, in order along the X-axis.

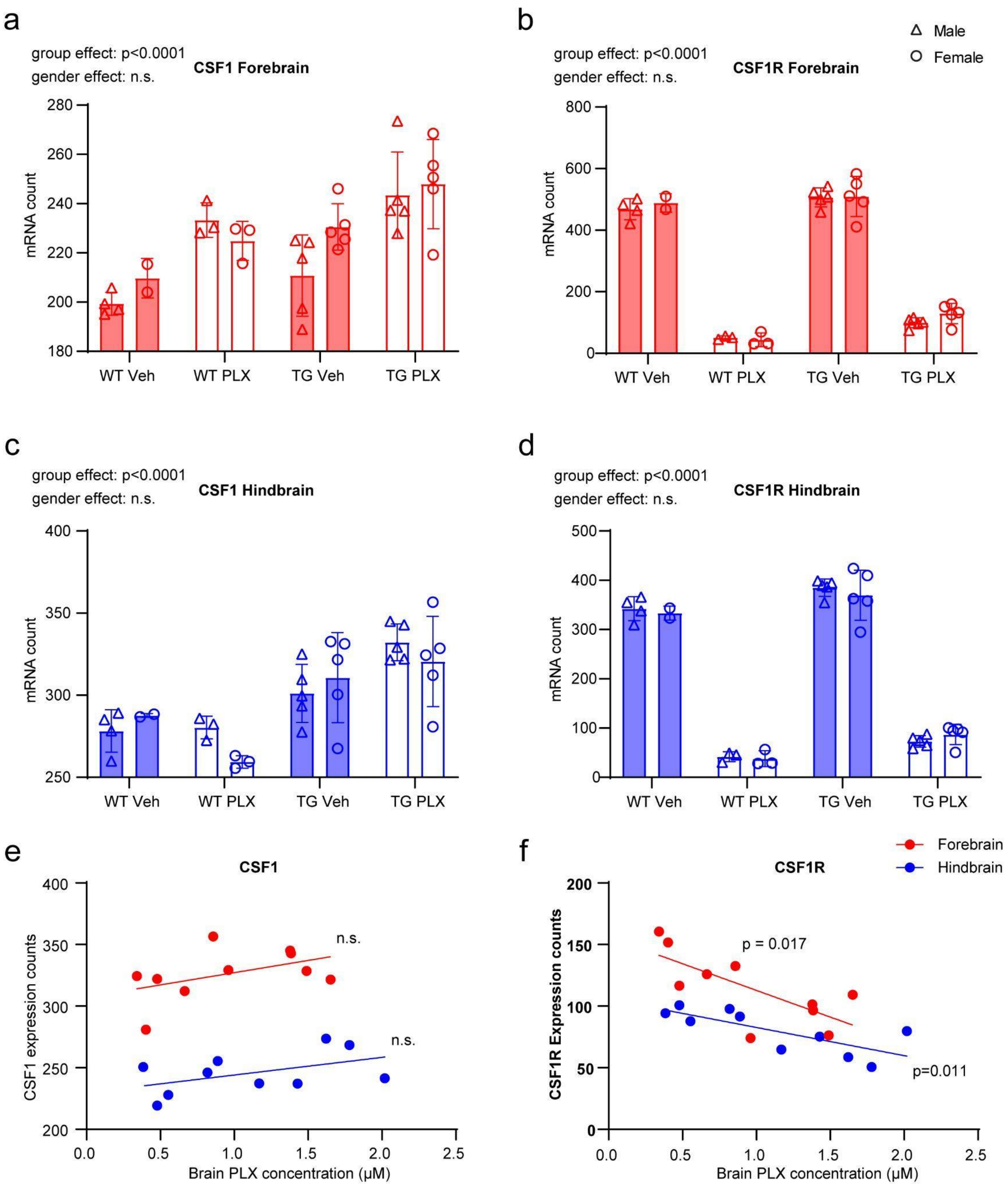

Supplementary Fig. 13| PLX treatment led to increased CSF1 expression and decreased CSF1R expression. Quantification of CSF1 expression showed slight increase after PLX treatment, while CSF1R expression showed marked decrease. Two-way ANOVA was used for statistical tests. Bottom panels showed scatter plots of brain PLX concentration against CSF1 or

CSF1R expression. Linear regressions were calculated for each group. In **a**–**d**, Data are presented as mean ± S.D., with n=4, 2, 3, 3, 5, 5, 5, 5 mice for each group, in order along the X-axis.

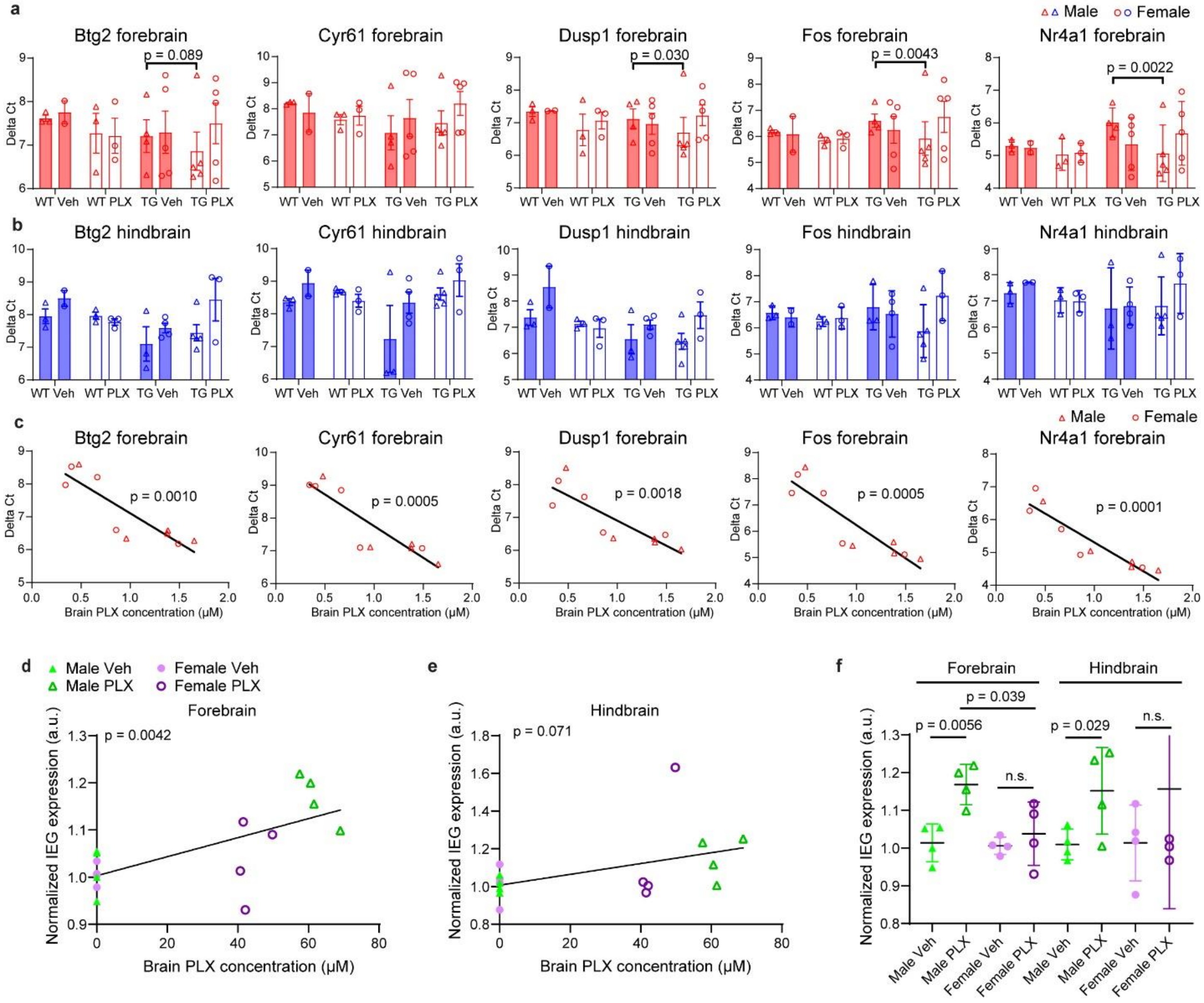

Supplementary Fig. 14| Validation of IEG up-regulation following PLX treatment. a, **b**, Real-time PCR quantifications of the mRNA levels of five IEGs in forebrains (**a**) and hindbrains (**b**) from PLX5622- or vehicle-treated Tg2541 or wildtype mice. **c,** Correlation of brain PLX concentration and expression levels from the five immediate early genes in the forebrains from PLX5622-treated Tg2541 mice. **c**, **d**, Correlation of brain PLX concentration and normalized expression levels from all immediate early genes (IEGs, 56 genes for each mouse) in the (**d**) forebrains and (**e**) hindbrains of vehicle- and PLX3397-treated Tg2541 mice. Pearson's correlation analysis was performed and the *P* values are shown. **f**, Quantification of normalized expression levels from all IEGs (56 genes for each mouse) in the forebrain and hindbrain of vehicle- and PLX3397-treated Tg2541 mice. Mann-Whitney tests were used for comparisons between groups. Each symbol represents an individual mouse. In **a** and **b**, data are presented as mean ± S.D., with n=4, 2, 3, 3, 5, 5, 5, 5 mice for each group, in order along the X-axis. In **f**, data are presented as mean ± S.D., with n=4 mice for each group.

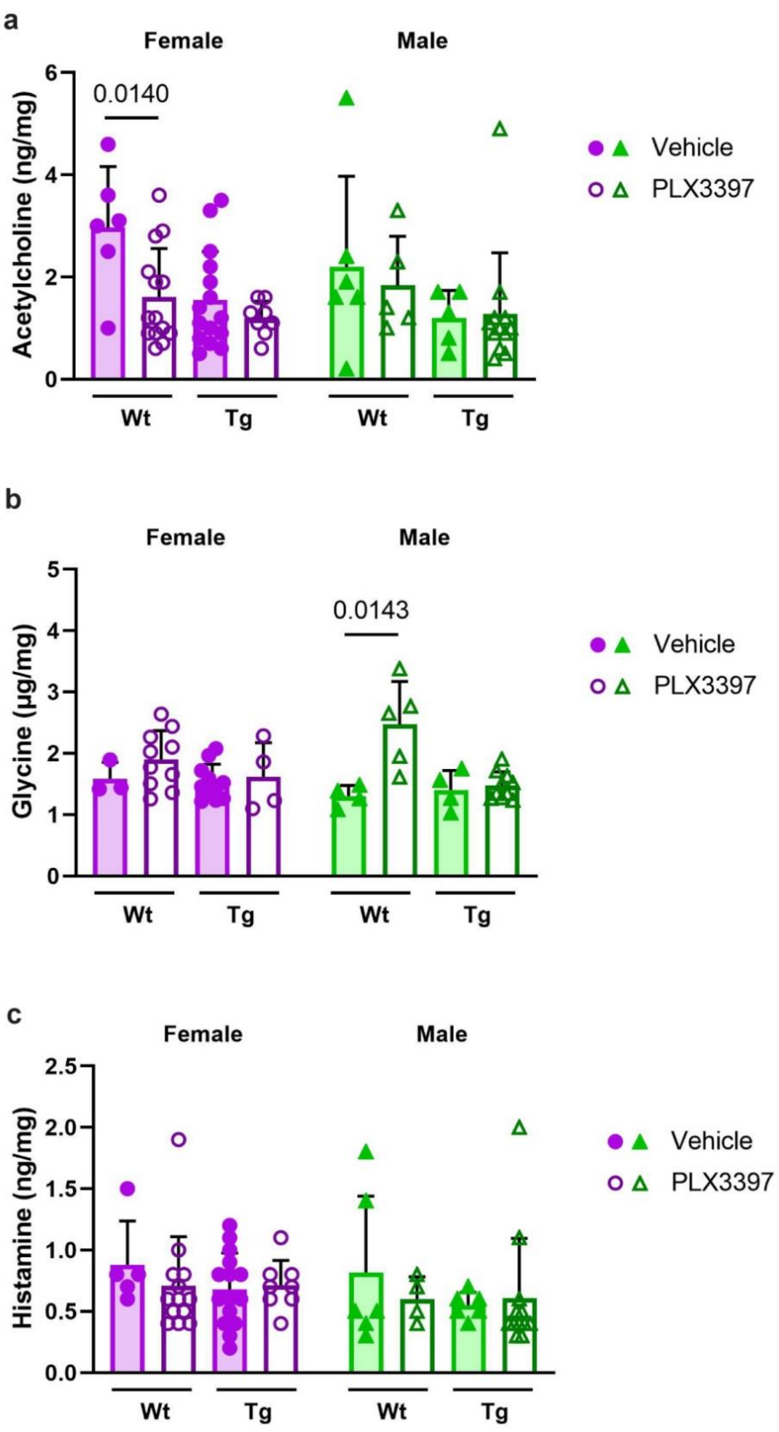

Supplementary Fig. 15| Brain neurotransmitter levels unaffected by PLX3397 in male Tg2541 mice. a, Forebrain lysates of wild type (Wt) or Tg2541 (Tg) mice, dosed for 6-12 weeks with PLX3397 (275 mg/kg oral) or vehicle beginning at 4 months of age, were measured by hydrophilic interaction liquid chromatography tandem mass spectrometry (HILIC-MS/MS). **b**,

HILIC-MS/MS analysis of the same samples for glycine. **c**, HILIC-MS/MS analysis of the same samples for histamine. **a−c,** Mann-Whitney tests were used for comparing vehicle and PLX3397 treatment groups within mouse genotype and sex, and the *P* values of statistically significant differences ($P < 0.05$) are shown. Each symbol represents an individual mouse and error bars indicate the s.d. of the mean. Raw data files from all metabolomics analyses are provided as Supplementary Data File 3.

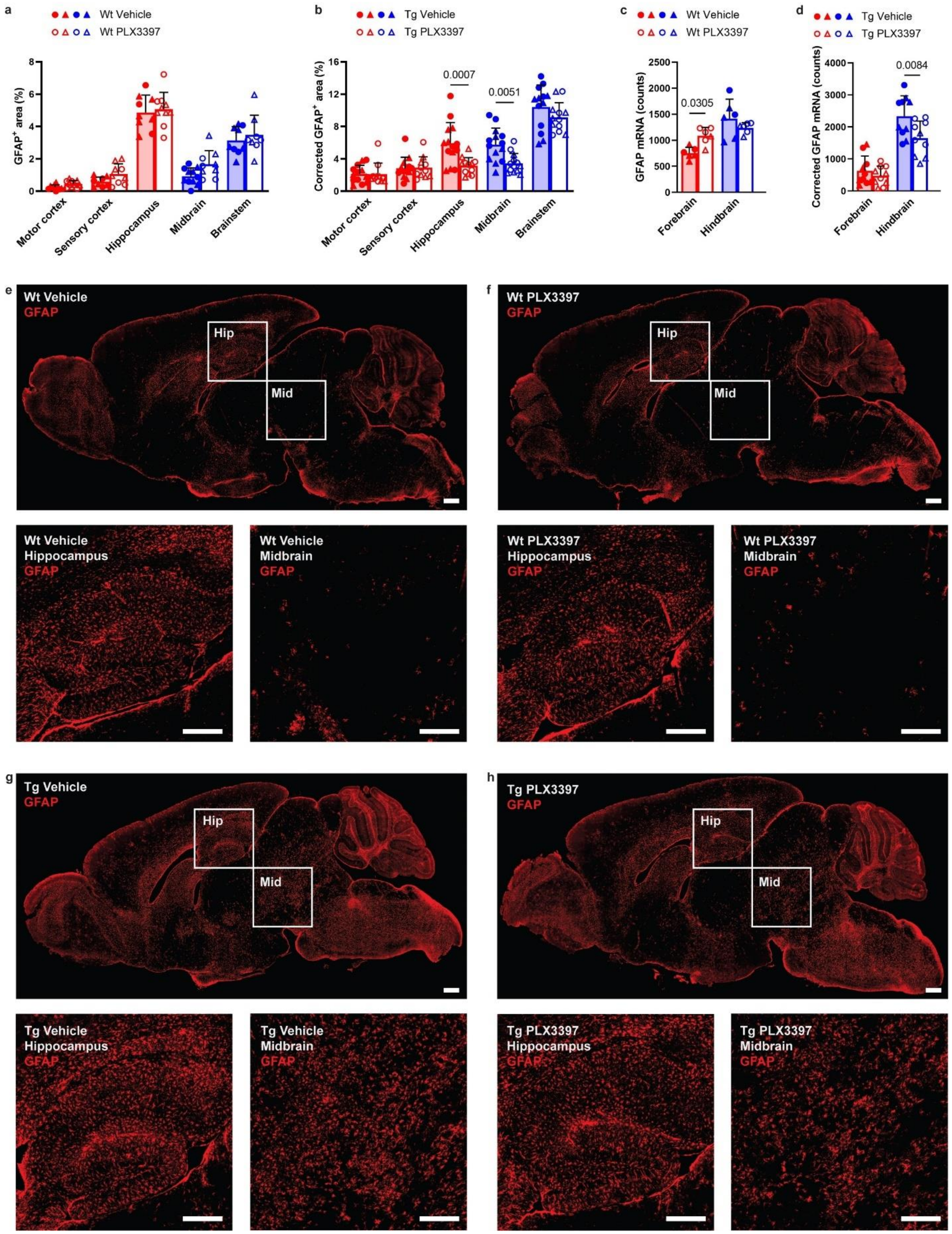

Supplementary Fig. 16| GFAP expression is reduced by PLX3397 treatment in Tg2541 mice. a, **b**, GFAP levels measured by IHC in five different brain regions, three forebrain regions

and two hindbrain regions, of (**a**) wild type (Wt) and (**b**) Tg2541 mice treated with PLX3397 (275 mg/kg oral) or vehicle. GFAP levels in Tg2541 mice were corrected for the average levels in each brain region of PLX3397- and vehicle-treated Wt mice. **c**, **d**, *GFAP* mRNA levels measured by Nanostring in the forebrain or hindbrain of (**c**) wild type and (**d**) Tg2541 mice treated with PLX3397 (275 mg/kg oral) or vehicle. *GFAP* mRNA levels in Tg2541 mice were corrected for the average levels in each brain region of PLX3397- and vehicle-treated Wt mice. In **a−d**, Two-way ANOVA with Holm-Šidák post hoc testing was used to evaluate the differences between vehicle- and PLX3397-treated groups within each brain region and *P* values of statistically significant differences ($P < 0.05$) are shown. Each symbol represents an individual mouse with female mice shown as closed or open circles and male mice shown as closed or open triangles. **e−h**, Representative GFAP IHC images of a brain section from (**e**) a Wt vehicle-treated mouse, (**f**) a Wt PLX3397-treated mouse, (**g**) a Tg2541 vehicle-treated mouse, and (**h**) a Tg2541 PLX3397-treated mouse. High-magnification images of the indicated hippocampus (Hip) and midbrain (Mid) regions are shown below each image. Scale bars, 500 µm.

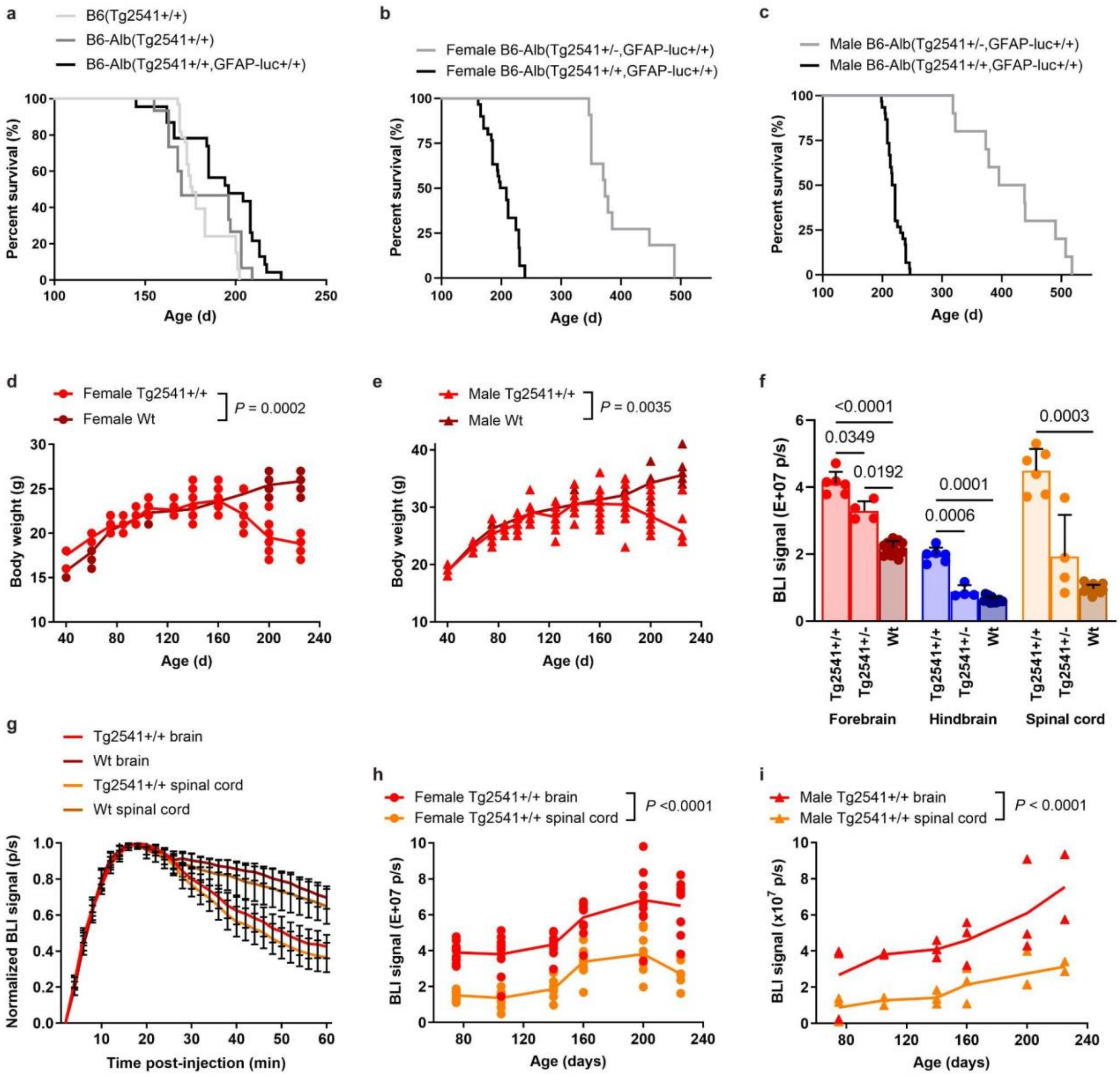

Supplementary Fig. 17| Generation, optimization, and validation of bigenic Tg2541/GFAP-luciferase mice for in vivo bioluminescent imaging (BLI) of astrocytosis. a, Kaplan-Meier plot shows that the survival curve (kinetics of disease) is unchanged for Tg2541 homozygous mice bred to B6-albino background and homozygous for the reporter GFAP-luciferase transgene. **b**, **c**, Kaplan-Meier plots for (**b**) female and (**c**) male mice showing that survival curves of homozygous and hemizygous B6-albino bigenic Tg2541 mice is not sex-dependent; homozygous Tg2541 mice have a median survival of 212 days, and hemizygous Tg2541 mice have a median survival of 378 days. **d**, **e**, As a crude surrogate of general health, longitudinal measurements of mouse body weight (grams) shows that in contrast to Wt mice, (**d**) female and (**e**) male Tg2541 mice lose weight as a result of decreased food intake from increasing

paraparesis with disease progression. Because earlier studies gave a standard volume of d-luciferin substrate regardless of changes in individual mouse weight, we optimized the protocol to give a 25mg/kg of CycLuc1 to increase consistency in BLI measurements. **f**, To validate the GFAP-luciferase reporter gene and the synthetic luciferin substrate (CycLuc1) *in vivo*, we performed BLI in Tg2541 homozygous mice (~200 days old) with advanced disease pathology and showed the BLI signal is significantly increased in the forebrain, hindbrain and spinal cord as compared to similar aged Tg2541 hemizygous and Wt mice. Welch ANOVA with Dunnett T3 post hoc testing was used to compare groups and *P* values for all statistically significant differences ($P < 0.05$) are shown. Error bars represent the s.d. of the mean. **g**, Time-lapse imaging (two-minute intervals) of BLI signal from brain and spinal cord in Tg and Wt mice showed that peak BLI signal occurred between 14 and 20 minutes after CycLuc1 injection to the peritoneum. Thus, to decrease variability in our study, we collect images at 14, 16, and 18 minutes post-injection and average all three time points to account for subtle differences in time of injection and individual mouse pharmacokinetic of CycLuc1. **h**, **i**, Longitudinal BLI plots in (**h**) female and (**i**) male mice show kinetics of gliosis in the Tg2541 brain and spinal cord (3 mice per field of view). **j**, Example image of the field of view (magnification) used to capture both the brain and spinal cord in three mice per time point. In **d**, **e**, **h**, and **i**, differences between groups were evaluated by repeated measures ANOVA and *P* values are shown. Each symbol represents an individual mouse and lines indicate group means.

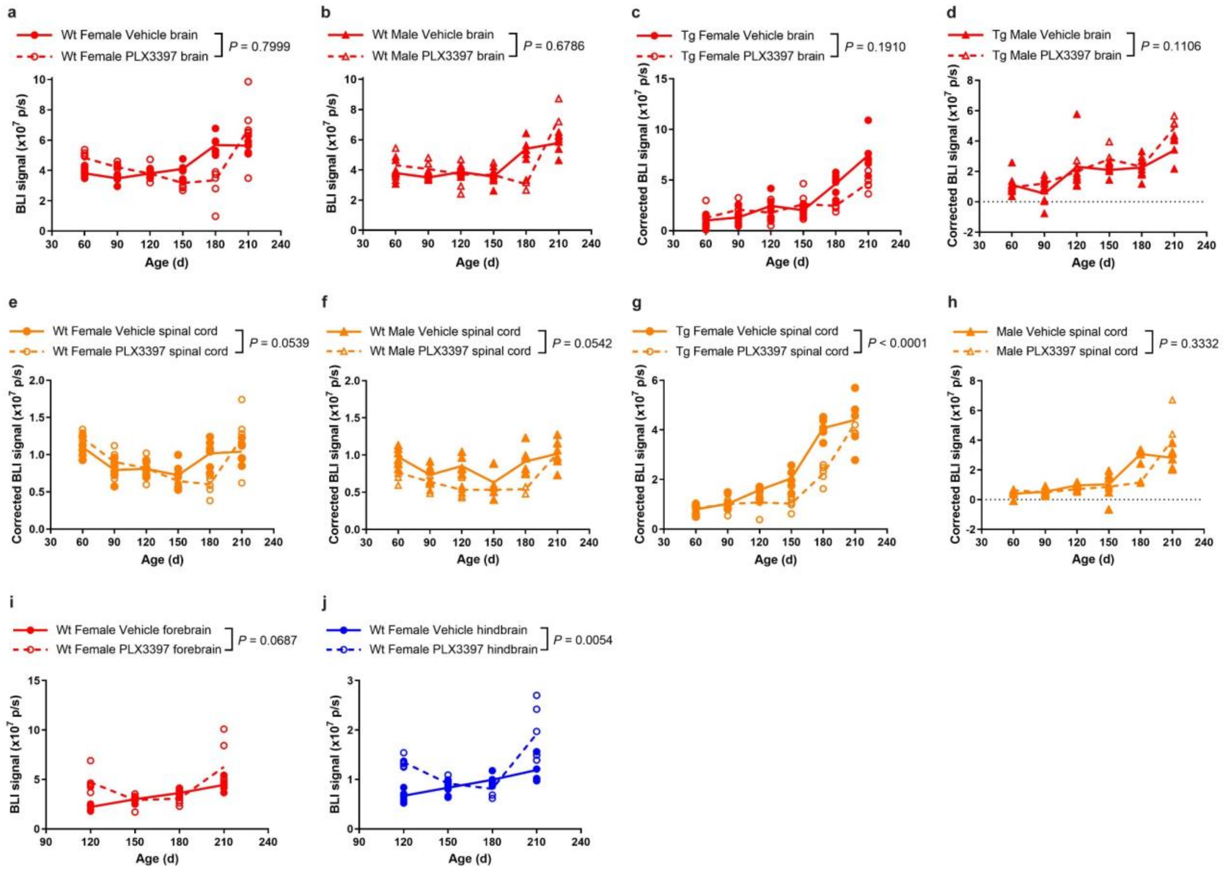

Supplementary Fig. 18| Brain and spinal cord BLI of PLX3397-treated wild type and Tg2541 mice. a, **b**, Longitudinal brain BLI plots of (**a**) female and (**b**) male wild type (Wt) mice treated with PLX3397 (275 mg/kg oral) or vehicle. **c**, **d**, Longitudinal brain BLI plots of (**c**) female and (**d**) male Tg2541 mice (Tg) treated with PLX3397 (275 mg/kg oral) or vehicle. The data were corrected for brain BLI levels of PLX3397- and vehicle-treated Wt mice, shown in **a** and **b**. Due to resolution and/or sensitivity of this magnification, we did not observe any differences between PLX3397 and vehicle groups. Higher magnification imaging on single mice from the same cohort was performed as shown in Fig. 8e−g. **e**, **f**, Longitudinal spinal cord BLI plots of (**e**) female and (**f**) male Wt mice treated with PLX3397 (275 mg/kg oral) or vehicle. **g**, **h**, Longitudinal spinal cord BLI plots of (**g**) female and (**h**) male Tg2541 mice (Tg) treated with PLX3397 (275 mg/kg oral) or vehicle. The data were corrected for spinal cord BLI levels of PLX3397- and vehicle-treated Wt mice, shown in **e** and **f**. **i**, **j**, High magnification BLI plots of the (**i**) forebrain and (**j**) hindbrain of female Wt mice treated with PLX3397 (275 mg/kg oral) or vehicle, and used to correct the BLI data of female Tg2541 mice presented in Fig. 8f,g. In **a−j**, differences between vehicle and PLX3397 treatment were evaluated by repeated measures ANOVA and *P* values are shown. Each symbol represents an individual mouse and lines

indicate group means.

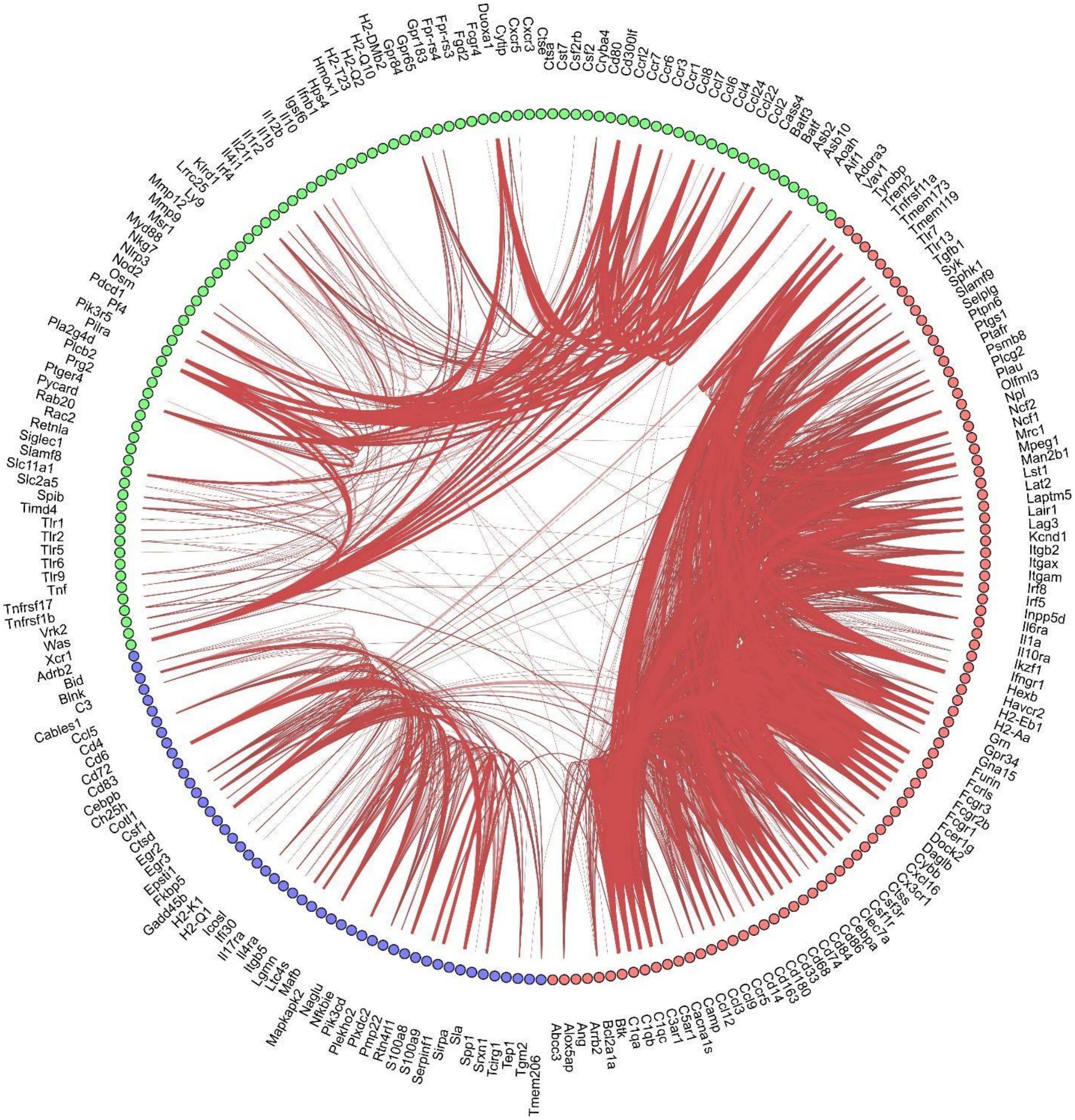

Supplementary Fig. 19| Clustering of microglia specific genes. The schemaball graph of microglial-specific genes as shown in Fig. 10. The name of each gene is shown next to the dot.

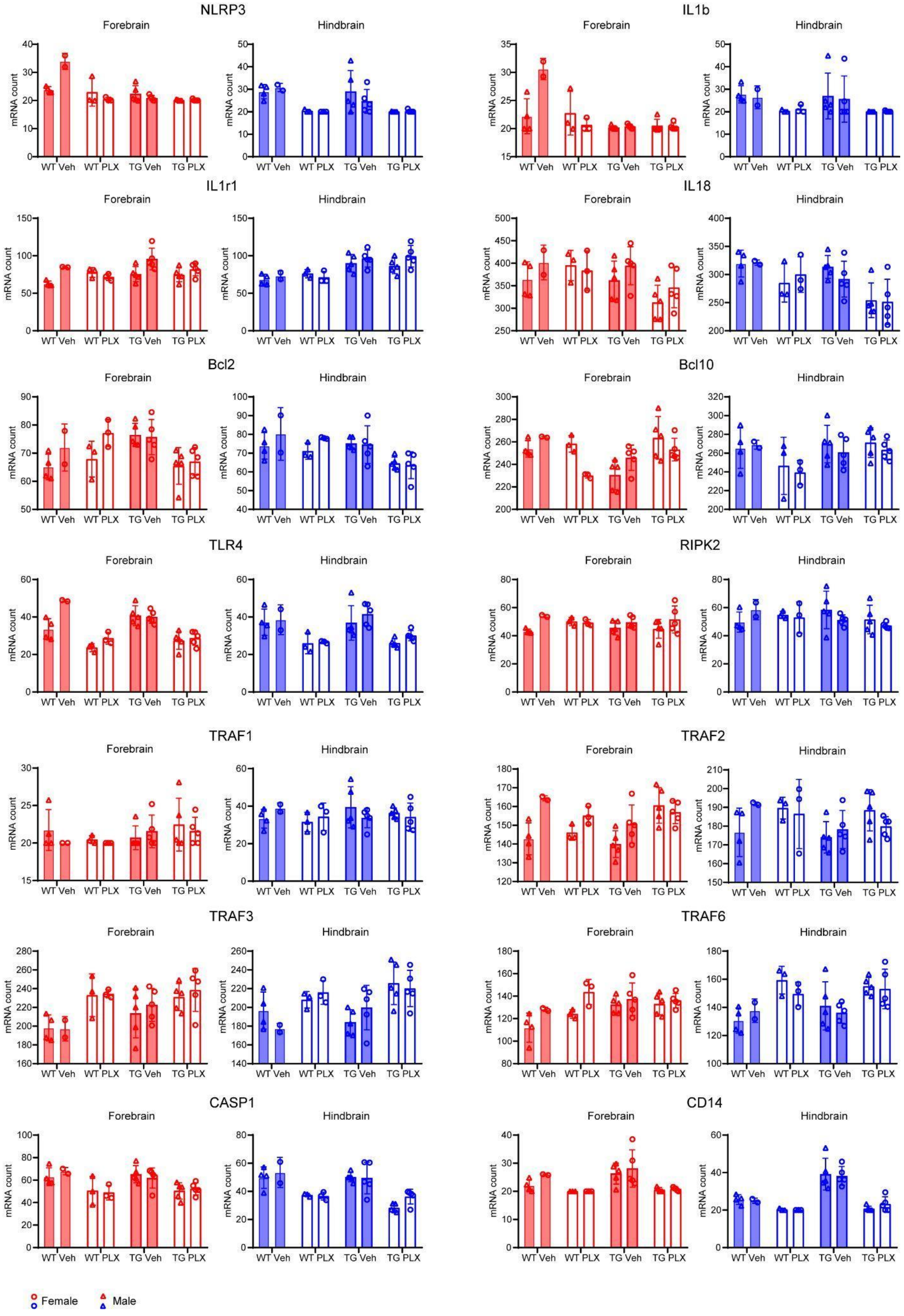
NLRP3
Forebrain
Hindbrain
IL1b
Forebrain
Hindbrain
IL1r1
Forebrain
Hindbrain
IL18
Forebrain
Hindbrain
Bcl2
Forebrain
Hindbrain
Bcl10
Forebrain
Hindbrain
TLR4
Forebrain
Hindbrain
RIPK2
Forebrain
Hindbrain
TRAF1
Forebrain
Hindbrain
TRAF2
Forebrain
Hindbrain
TRAF3
Forebrain
Hindbrain
TRAF6
Forebrain
Hindbrain
CASP1
Forebrain
Hindbrain
CD14
Forebrain
Hindbrain
mRNA count
WT Veh
WT PLX
TG Veh
TG PLX
Female
Male

Supplementary Fig. 20| Tau pathology or PLX treatment did not lead to marked alteration of inflammasome-related genes. Quantification of 14 inflammasome-related genes in different experimental groups. For each graph, data are presented as mean ± S.D., with n=4, 2, 3, 3, 5, 5, 5, 5 mice for each group, in order along the X-axis.
