# Supplementary material for: CSF1R inhibitors induce a sex-specific resilient microglial phenotype and functional rescue in a tauopathy mouse model
Source: Nat Commun. 2023 Jan 9;14:118. doi: 10.1038/s41467-022-35753-w (PMC9829908; doi:10.1038/s41467-022-35753-w)
Supplement: Supplementary file 3 — Description of Additional Supplementary Files [file 41467_2022_35753_MOESM3_ESM.pdf]

Supplementary Data File 1| Statistical tests of sex-specific drug effects on microglia, tau, and neurons. **a**, Statistical test results for 3-way ANOVA analyses for each figure panel where female and male mice were combined. Statistical test results include the type III sum-of-squares (SS), the F statistic, and the *P* value for the main effect of sex and for the sex*drug interaction effect. **b**, **c**, Statistical test results for 2-way ANOVA analyses for (**b**) female or (**c**) male mice separately, for each figure panel where female and male mice were combined. Statistical test results include the type III sum-of-squares (SS), the F statistic, and the *P* value for the main effect of drug, and the *P* value for the multiple comparisons post-hoc testing of each drug compared to the vehicle in the forebrain, hindbrain, or spinal cord. 'na' indicates that the data is not available because the drug was not tested in that particular experiment.

Supplementary Data File 2| Differentially expressed genes in male and female PLX-treated Tg2541 mice. Forebrain samples from male and female Tg2541 mice were analyzed for gene expression by Nanostring following chronic treatment with PLX5622. The data represent the average expression levels of five male and five female mice, which were then compared by unpaired t-test and the *P* value is shown. Please note that the transcriptome (Nanostring) data generated in this study which includes data from hindbrain, wild type, and vehicle-treated mice have been deposited in the Zenodo database under accession code 7415371 [https://doi.org/10.5281/zenodo.7415371].

Supplementary Data File 3| Mass spectrometry data for metabolomics analyses. Data supporting metabolomics analyses in Fig. 7 and Supplementary Fig. 15 are provided. Sex and group allocation for each mouse are listed and associated with a unique file name in the raw dataset. The raw mass spectrometry data (raw output files from Multiquant software) generated in this study have been deposited in the Zenodo database under accession code 7415371 [https://doi.org/10.5281/zenodo.7415371].
