# Supplementary material for: CSF1R inhibitors induce a sex-specific resilient microglial phenotype and functional rescue in a tauopathy mouse model
Source: Nat Commun. 2023 Jan 9;14:118. doi: 10.1038/s41467-022-35753-w (PMC9829908; doi:10.1038/s41467-022-35753-w)
Supplement: Supplementary file 7 — Reporting Summary [file 41467_2022_35753_MOESM7_ESM.pdf]

nature portfolio

Corresponding author(s): Carlo Condello

Last updated by author(s): Dec 8, 2022

Reporting Summary

Nature Portfolio wishes to improve the reproducibility of the work that we publish. This form provides structure for consistency and transparency in reporting. For further information on Nature Portfolio policies, see our Editorial Policies and the Editorial Policy Checklist.

Statistics

For all statistical analyses, confirm that the following items are present in the figure legend, table legend, main text, or Methods section.

n/a	Confirmed	
☐	☒	The exact sample size (*n*) for each experimental group/condition, given as a discrete number and unit of measurement
☐	☒	A statement on whether measurements were taken from distinct samples or whether the same sample was measured repeatedly
☐	☒	The statistical test(s) used AND whether they are one- or two-sided<br>*Only common tests should be described solely by name; describe more complex techniques in the Methods section.*
☒	☐	A description of all covariates tested
☐	☒	A description of any assumptions or corrections, such as tests of normality and adjustment for multiple comparisons
☐	☒	A full description of the statistical parameters including central tendency (e.g. means) or other basic estimates (e.g. regression coefficient) AND variation (e.g. standard deviation) or associated estimates of uncertainty (e.g. confidence intervals)
☐	☒	For null hypothesis testing, the test statistic (e.g. *F*, *t*, *r*) with confidence intervals, effect sizes, degrees of freedom and *P* value noted<br>*Give P values as exact values whenever suitable.*
☒	☐	For Bayesian analysis, information on the choice of priors and Markov chain Monte Carlo settings
☒	☐	For hierarchical and complex designs, identification of the appropriate level for tests and full reporting of outcomes
☐	☒	Estimates of effect sizes (e.g. Cohen's *d*, Pearson's *r*), indicating how they were calculated

Our web collection on statistics for biologists contains articles on many of the points above.

Software and code

Policy information about availability of computer code

Data collection	Immunohistochemical imaging data were collected using Zeiss Zen 2.3 software. Tau-prion cellular bioassay data were collected using GE Healthcare INCell Analyzer 6000 software. Brain homogenate total protein data were collected using Biotek Gen5 v3.0 software. Mouse home-cage monitoring data were collected using the Sable Systems International Promethion MetaScreen software package. Drug pharmacokinetic data were collected using SCIEX MultiQuant Software. SIMOA data were collected using Quanterix HD-1 Analyzer software. Nanostring RNA expression data were collected using Nanostring nSolver 4.0 software. Gene expression data by RT-qPCR were collected using Applied Biosystems QuantStudio Design and Analysis v2.5 software. In vivo bioluminescence imaging data were collected using PerkinElmer Living Image v4.4 software. Confocal microscopy imaging data were collected using Leica Application Suite X software.
Data analysis	Immunohistochemical imaging data were analyzed using Zeiss Zen 2.3 software. Gene expression pattern data and confocal imaging data were analyzed using custom MATLAB code. Pathway analysis was performed using Ingenuity Pathway Analysis software v22.0 (QIAGEN). Metabolomics data was analyzed using free, publicly-available MS-DIAL software (Tsugawa et al. 2015, Nat Methods, 12:523-526). All statistical analyses were performed using GraphPad Prism 8.0 software.

For manuscripts utilizing custom algorithms or software that are central to the research but not yet described in published literature, software must be made available to editors and reviewers. We strongly encourage code deposition in a community repository (e.g. GitHub). See the Nature Portfolio guidelines for submitting code & software for further information.

Data

Policy information about availability of data

All manuscripts must include a data availability statement. This statement should provide the following information, where applicable:

- Accession codes, unique identifiers, or web links for publicly available datasets
- A description of any restrictions on data availability
- For clinical datasets or third party data, please ensure that the statement adheres to our policy

The authors declare that all data supporting the findings of this study are available within the paper and its supplementary information files. The transcriptome data (Nanostring) that support the findings of this study, specifically in Figures 6-9, are available in Github with the following link: https://gitfront.io/r/user-8849465/665dd65fd9d9e78650ed02b9f30236d99240de39/UCSF-PLX-nanostring/.

Human research participants

Policy information about studies involving human research participants and Sex and Gender in Research.

Reporting on sex and gender	*Use the terms sex (biological attribute) and gender (shaped by social and cultural circumstances) carefully in order to avoid confusing both terms. Indicate if findings apply to only one sex or gender; describe whether sex and gender were considered in study design whether sex and/or gender was determined based on self-reporting or assigned and methods used. Provide in the source data disaggregated sex and gender data where this information has been collected, and consent has been obtained for sharing of individual-level data; provide overall numbers in this Reporting Summary. Please state if this information has not been collected. Report sex- and gender-based analyses where performed, justify reasons for lack of sex- and gender-based analysis.*
Population characteristics	*Describe the covariate-relevant population characteristics of the human research participants (e.g. age, genotypic information, past and current diagnosis and treatment categories). If you filled out the behavioural & social sciences study design questions and have nothing to add here, write "See above."*
Recruitment	*Describe how participants were recruited. Outline any potential self-selection bias or other biases that may be present and how these are likely to impact results.*
Ethics oversight	*Identify the organization(s) that approved the study protocol.*

Note that full information on the approval of the study protocol must also be provided in the manuscript.

Field-specific reporting

Please select the one below that is the best fit for your research. If you are not sure, read the appropriate sections before making your selection.

☒ Life sciences ☐ Behavioural & social sciences ☐ Ecological, evolutionary & environmental sciences

For a reference copy of the document with all sections, see nature.com/documents/nr-reporting-summary-flat.pdf

Life sciences study design

All studies must disclose on these points even when the disclosure is negative.

Sample size	Sample sizes for all experiments were selected prospectively based on our previous work with Tg2541 mice (Johnson et al., 2017, Proc Natl Acad Sci USA; Aoyagi et al., 2019, Sci Transl Med).
Data exclusions	No data were excluded from the analyses.
Replication	Most of the mouse dosing studies were replicated at least twice at different times, the replications were confirmed to be successful, and the data from the studies were combined.
Randomization	In all experiments, mice were randomly assigned to groups and sex was balanced as best possible given the mice available.
Blinding	In all experiments, investigators were blinded to the group allocation of mice while performing experiments or analyzing samples.

Reporting for specific materials, systems and methods

We require information from authors about some types of materials, experimental systems and methods used in many studies. Here, indicate whether each material, system or method listed is relevant to your study. If you are not sure if a list item applies to your research, read the appropriate section before selecting a response.

Materials & experimental systems

n/a	Involved in the study
☐	☒ Antibodies
☐	☒ Eukaryotic cell lines
☒	☐ Palaeontology and archaeology
☐	☒ Animals and other organisms
☒	☐ Clinical data
☒	☐ Dual use research of concern

Methods

n/a	Involved in the study
☒	☐ ChIP-seq
☒	☐ Flow cytometry
☒	☐ MRI-based neuroimaging

Antibodies

Antibodies used

Primary antibodies: rabbit monoclonal anti-Iba1 (Abcam, ab178847), rabbit polyclonal anti-P2yr12 (Atlas, HPA014518), mouse monoclonal anti-NeuN (Millipore, MAB377, clone A60), chicken polyclonal anti-GFAP (Abcam, ab4674), mouse monoclonal anti-pS202/T205 tau (Thermo Fisher, MN1020, clone AT8), and mouse monoclonal anti-CD206 (Biorad, MCA2235, clone MR5D3), all used at 1:250 dilution. Secondary antibodies: Goat anti-mouse IgG (H+L) highly cross-adsorbed secondary antibody conjugated to Alexa Fluor Plus 488 (A48286), 555 (A48287), or 647 (A32728); Goat anti-rabbit IgG (H+L) highly cross-adsorbed secondary antibody conjugated to Alexa Fluor Plus 488 (A32731), 555 (A32732), or 647 (A32733); Goat anti-chicken IgG (H+L) highly cross-adsorbed secondary antibody conjugated to Alexa Fluor Plus 488 (A32931), 555 (A32932), or 647 (A32933), all from Life Technologies and all used at 1:500 dilution.

Validation

All antibodies are commercially available, commonly used in the field and validated for immunohistochemistry by both manufacturer and our lab. We have optimized each antibody for use in fluorescence immunohistochemistry experiments, and have been used in previous peer-reviewed studies such as: Yuan et al., J. Neurosci. 2016; Yuan et al., Neuron. 2016; Johnson et al., PNAS 2017. Some antibodies are cross-reactive across multiple species, but in this study we employ them in transgenic mice only and thus no species validation is necessary. NeuN labels neuronal nuclei, and we confirmed cell-specificity in mice in validation experiments by using in combination with markers of other neural cells such as microglia (Iba1), astrocyes (Gfap), and oligodendrocytes (Olig2). We also performed similar validation experiments to confirm the specificity of Iba1 and P2ry12 in mice, proteins specific to microglia in the brain.

Eukaryotic cell lines

Policy information about cell lines and Sex and Gender in Research

Cell line source(s)

Human embryonic kidney (HEK293T) cells were purchased from ATCC.

Authentication

Following transfection of HEK293T cells to express the repeat domain of 4R human tau (aa 243–375) containing the P301L and V337M mutations and C-terminally fused to YFP, as previously described (Kfoury et al., 2012, J Biol Chem; Sanders et al., 2014, Neuron), the cell line was authenticated by immunoblotting with antibodies directed against the repeat domain of human tau, and by immunofluorescence imaging of the YFP-tagged tau, which aggregated intracellularly in response to exposure to tau-prion species.

Mycoplasma contamination

The HEK293T cell line was tested and found to be negative for mycoplasma contamination.

Commonly misidentified lines
(See ICLAC register)

No commonly misidentified cell lines were used in the study.

Animals and other research organisms

Policy information about studies involving animals; ARRIVE guidelines recommended for reporting animal research, and Sex and Gender in Research

Laboratory animals

Tg2541 transgenic mice on a mixed C57BL/6J x CBA/Ca background were kindly provided by Dr. Michel Goedert (Medical Research Council, UK), and were bred onto a congenic C57BL/6J background by marker-assisted backcrossing with C57BL/6J wildtype mice, purchased from The Jackson Laboratory. Both male and female Tg2541 mice were used at 2 or 4 months of age for dosing experiments, or 10 weeks of age for stereotaxic inoculation experiments. Albino C57BL/6J for bioluminescence imaging experiments were purchased from The Jackson Laboratory (strain no. 000058). Tg(Gfap-luc) mice for bioluminescence imaging experiments were a gift from Caliper Life Sciences (now Perkin-Elmer). Both male and female albino C57BL/6J mice and Tg(Gfap-luc) mice were used at the same ages described above for the respective experiments. Mice were housed with a 14h light/10h dark cycle with an average ambient temperature of 24C and an average humidity of 61%.

Wild animals

The study did not involve wild animals.

Reporting on sex

Sex was considered in the experimental design. Source data has been provided disaggregated for sex, and sex is clearly differentiated in the figures. Sex-based analyses were performed and are described throughout the paper.

Field-collected samples	The study did not involve field-collected samples.
Ethics oversight	All procedures for animal use were approved by the University of California, San Francisco's Institutional Animal Care and Use Committee.

Note that full information on the approval of the study protocol must also be provided in the manuscript.
